# YTHDF1 loss in dendritic cells potentiates radiation-induced antitumor immunity via STING-dependent type I IFN production

**DOI:** 10.1172/JCI181612

**Published:** 2024-12-02

**Authors:** Chuangyu Wen, Liangliang Wang, András Piffkó, Dapeng Chen, Xianbin Yu, Katarzyna Zawieracz, Jason Bugno, Kaiting Yang, Emile Z. Naccasha, Fei Ji, Jiaai Wang, Xiaona Huang, Stephen Y. Luo, Lei Tan, Bin Shen, Cheng Luo, Megan E. McNerney, Steven J. Chmura, Ainhoa Arina, Sean Pitroda, Chuan He, Hua Laura Liang, Ralph R. Weichselbaum

**Affiliations:** 1 Department of Radiation and Cellular Oncology and; 2 Ludwig Center for Metastasis Research, University of Chicago, Chicago, Illinois, USA.; 3 The Laboratory of Microbiome and Microecological Technology, Institute of Microbiology, Chinese Academy of Sciences, Beijing, China.; 4 Department of Neurosurgery, University Medical Center Hamburg-Eppendorf, Hamburg, Germany.; 5 Department of Chemistry, Department of Biochemistry and Molecular Biology, and Institute for Biophysical Dynamics,; 6 Howard Hughes Medical Institute, and; 7 Department of Pathology, University of Chicago, Chicago, Illinois, USA.; 8 Biomedical Engineering Program, Northwestern University, Evanston, Illinois, USA.; 9 State Key Laboratory of Reproductive Medicine and Offspring Health, Women’s Hospital of Nanjing Medical University, Nanjing Women and Children’s Healthcare Hospital, Center for Global Health, Gusu School, Nanjing Medical University, Nanjing, Jiangsu, China.; 10 State Key Laboratory of Drug Research, Shanghai Institute of Materia Medica, Chinese Academy of Sciences, Shanghai, China.; 11 Zhongshan Institute for Drug Discovery, Shanghai Institute of Materia Medica, Chinese Academy of Sciences, Zhongshan, Guangdong, China.

**Keywords:** Immunology, Oncology, Cancer immunotherapy, Dendritic cells, Radiation therapy

## Abstract

The RNA *N*^6^-methyladenosine (m^6^A) reader YTHDF1 is implicated in cancer etiology and progression. We discovered that radiotherapy (RT) increased YTHDF1 expression in dendritic cells (DCs) of PBMCs from patients with cancer, but not in other immune cells tested. Elevated YTHDF1 expression in DCs was associated with poor outcomes for patients receiving RT. We found that loss of *Ythdf1* in DCs enhanced the antitumor effects of ionizing radiation (IR) by increasing the cross-priming capacity of DCs across multiple murine cancer models. Mechanistically, IR upregulated YTHDF1 expression in DCs through stimulator of IFN genes/type I IFN (STING/IFN-I) signaling. YTHDF1 in turn triggered STING degradation by increasing lysosomal cathepsins, thereby reducing IFN-I production. We created a YTHDF1 deletion/inhibition prototype DC vaccine that significantly improved the therapeutic effect of RT and radioimmunotherapy in a murine melanoma model. Our findings reveal a layer of regulation between YTHDF1/m^6^A and STING in response to IR, which opens new paths for the development of YTHDF1-targeting therapies.

## Abstract 2

YTHDF1 elevation in DCs post-radiotherapy is associated with poor outcomes in patients. YTHDF1 deletion or inhibition in DCs enhances the antitumor effects of radiation. YTHDF1 negatively regulates the cross-priming capacity of DCs via impairing STING-IFN-I signaling.

## Graphical abstract

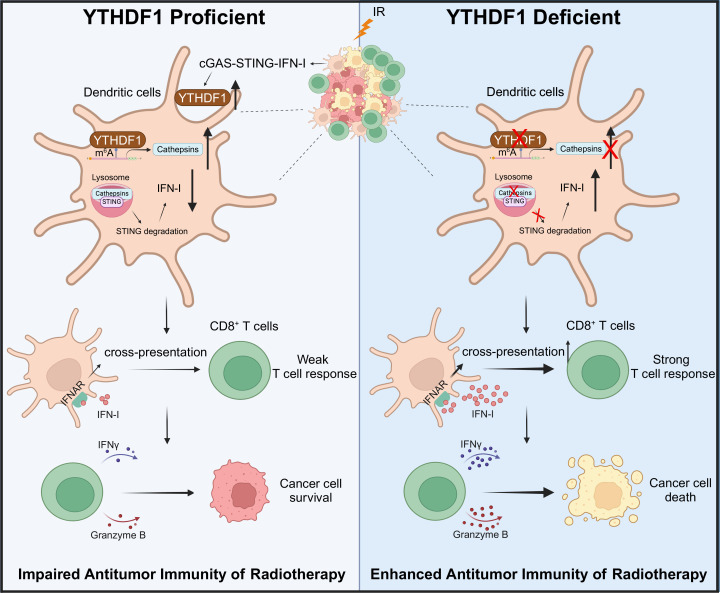

## Introduction

The YTH family *N*^6^-methyladenosine RNA-binding protein YTHDF1 is an *N*^6^-methyadenosine (m^6^A) reader protein and the binding of YTHDF1 to m^6^A-modified mRNA lead to an increase in translation of the target transcripts (1–5). YTHDF1 is implicated in driving tumorigenesis, with elevated YTHDF1 linked to poor outcomes in several cancer types including hepatocellular carcinoma, colorectal cancer, and acute myeloid leukemia (6–9). Limited data are available regarding the antitumor role of YTHDF1 in immune cells; the loss of *Ythdf1* in dendritic cells (DCs) has been reported to inhibit tumor growth in murine cancer models (10). Much remains to be elucidated regarding the function of YTHDF1 in immune cells, particularly in the context of radiotherapy (RT) and immunotherapy.

RT is used in more than 50% of patients with cancer and is commonly applied to achieve local tumor control (11, 12). More recently, RT has been used to treat metastatic disease with immunotherapy or with focused high-dose RT in oligometastatic disease (13–17). Accumulating evidence suggests that, following ionizing radiation (IR), increased antigen presentation by DCs is required to connect the innate and adaptive immune systems, which is critical to elicit the full antitumor effects of IR (18–24). YTHDF1 impedes the antigen presentation of DCs (10), suggesting a relationship between m^6^A/YTHDF1 and DC function. However, the effect of YTHDF1 on the antitumor effects of IR and the underlying mechanisms of action remain largely unknown.

Activation of stimulator of IFN genes (STING) leads to an increase of type I IFN (IFN-I) production in DCs, which is important for enhancing DC-mediated antitumor immunity (25–28). Our previous studies have demonstrated that IR exerts antitumor immunity via activating the STING-IFN-I signaling in DCs (18, 21). Several studies have reported the connection between YTHDF1 and IFN-I. However, these studies have shown conflicting results in different cancer cell lines (29, 30), suggesting that there is a complex relationship between YTHDF1 and IFN-I in different contexts, which could be critical to the development of new therapeutic strategies that involve modulation of DCs.

We report that RT-induced YTHDF1 expression in DCs correlated with poor progression-free survival in patients. In murine cancer models, IR activation of STING/IFN-I signaling induced YTHDF1 expression. Enhanced YTHDF1 production led to cathepsin-mediated degradation of STING protein, revealing what we believe to be a previously unknown regulation of STING through m^6^A and YTHDF1. *Ythdf1* deletion promoted IFN-I production in DCs and enhanced the cross-priming capacity of DCs and CD8^+^ T cell–mediated tumor killing in murine cancer models following IR. Moreover, administration of an experimental prototype DC vaccine using *Ythdf1*-deficient DCs or YTHDF1 inhibitor treatment improved the antitumor effect of both IR and IR plus anti–programmed death ligand 1 (anti–PD-L1) combination therapy. Our findings provide insights into the mechanisms by which STING/IFN-I/YTHDF1 negative feedback regulates the antitumor function of DCs and the associated antitumor immunity elicited by IR.

## Results

### Ythdf1 deficiency in DCs enhances the antitumor response to RT.

**Figure 1:**
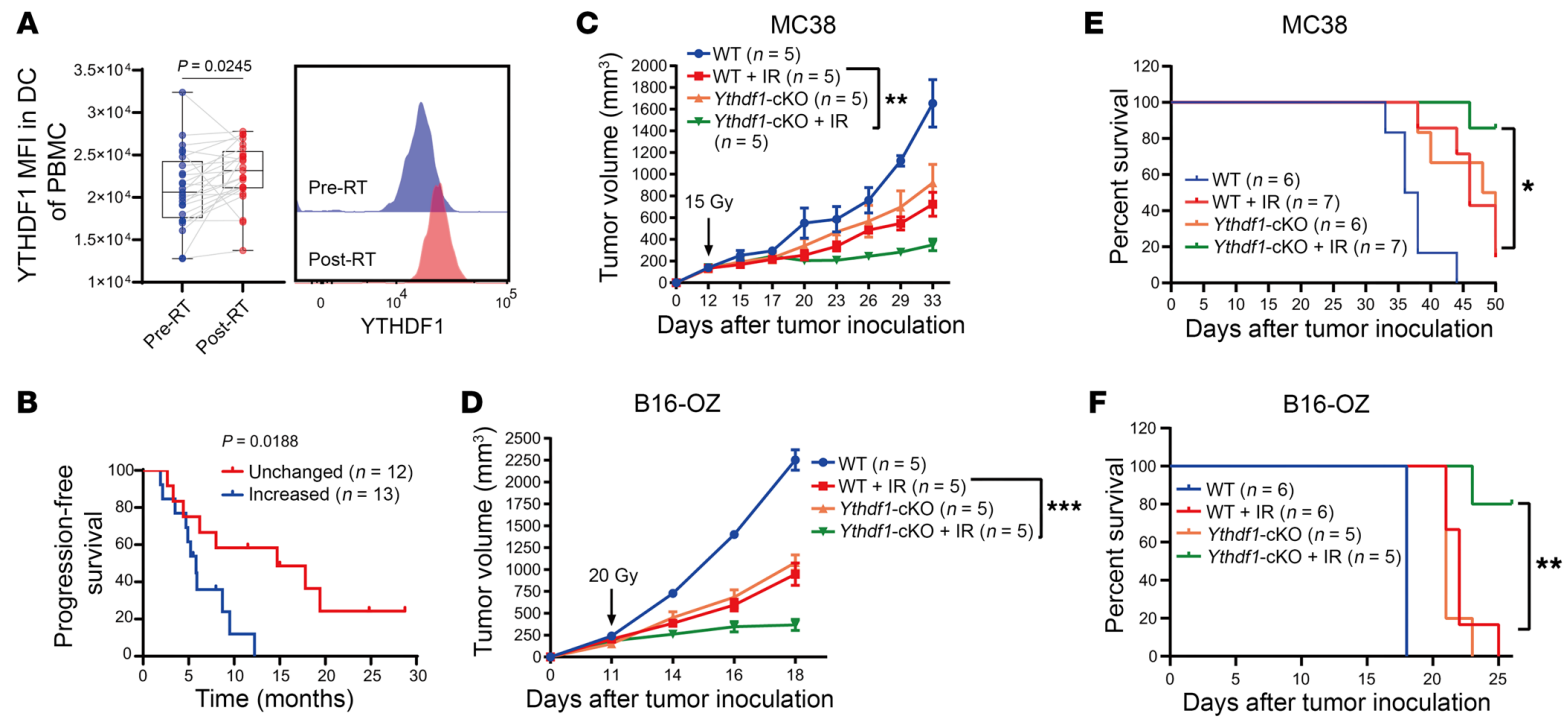
*Ythdf1* deficiency in DCs enhances the antitumor response to RT. (**A**) MFI of YTHDF1 in DCs (CD11c^+^HLA-DR^+^) of PBMCs from patients with metastatic NSCLC with SBRT treatment via flow cytometry (*n* = 25). (**B**) Kaplan-Meier analysis of progression-free survival according to increased and unchanged YTHDF1 expression in DCs of PBMCs from NSCLC patients with SBRT treatment. (**C**–**F**) WT (*Ythdf1^fl/fl^*) and *Ythdf1*-cKO (*Cd11c^Cre^* *Ythdf1^fl/fl^*) mice were injected s.c. with MC38 (**C** and **E**) and B16-OZ (**D** and **F**) cells. Tumor-bearing mice were treated with local IR (1 dose) when the tumor volume reached 100–200 mm^3^. Tumor growth (**C** and **D**) and survival (**E** and **F**) were monitored after IR. Mice were considered dead if tumor volumes reached 2,000 mm^3^. Data are presented as the mean ± SEM. Data are representative of 2 or 3 independent experiments (**C**–**F**). Two-sided, paired Student’s *t* test (**A**), 2-sided log-rank (Mantel-Cox) test (**B**, **E** and **F**), and 2-way ANOVA with Tukey’s multiple-comparison test (**C** and **D**). *P < 0.05, **P < 0.01, and ***P < 0.001.

To evaluate YTHDF1 expression in patients receiving RT, we measured YTHDF1 levels in PBMCs from patients with metastatic non–small cell lung cancer (NSCLC) enrolled in a clinical trial at our institution (COSINR study, NCT03223155); patients were treated with sequential or concurrent stereotactic body radiotherapy (SBRT) and immune checkpoint blockade therapy (31). Briefly, by high-dimensional flow cytometry, we that found YTHDF1 levels in DCs (CD11c^+^HLA-DR^+^ population) were significantly increased after RT compared with matched pre-RT samples (*P* = 0.0245, Figure 1A), whereas no significant changes were observed in other immune cell populations tested (Supplemental Figure 1; supplemental material available online with this article; https://doi.org/10.1172/JCI181612DS1). We examined the relationship between increased YTHDF1 expression and clinical outcomes. The median fold change in YTHDF1 expression after RT versus before RT was used to stratify patients into groups with increased or unchanged YTHDF1 expression. When we stratified patients by median fold changes of YTHDF1 expression, we found that increased YTHDF1 expression in DCs was associated with poor progression-free survival in these patients with NSCLC (*P* = 0.0188, Figure 1B). Together with previous preclinical results showing notable DC effects in *Ythdf1* whole-body KO studies (10), we hypothesized that RT-induced YTHDF1 expression in DCs may account for the weakened antitumor efficacy.

To investigate this hypothesis, we generated *Ythdf1^fl/fl^* transgenic mice (Supplemental Figure 2, A and B). Mice with DC-specific *Ythdf1* deletion were generated by breeding *Cd11c^Cre^* with *Ythdf1^fl/fl^* mice to create conditional KO mice (hereafter referred to as *Ythdf1*-cKO mice) (Supplemental Figure 2, C and D). *Ythdf1*-cKO and *Ythdf1^fl/fl^* (WT) mice were used to evaluate the antitumor effects of DC-specific *Ythdf1* deletion in murine cancer models. The mice were treated with local tumor single high-dose IR (analogous to doses used in clinical SBRT). IR resulted in a pronounced inhibition of tumor growth in *Ythdf1*-cKO mice compared with WT mice, as assessed by both tumor volume and animal survival, in both MC38 colon carcinoma and B16-SIINFEKL(OT-I)-Zsgreen (B16-OZ) melanoma models (Figure 1, C–F). We observed similar results in *Ythdf1* whole-body KO (*Ythdf1*-KO) mice (Supplemental Figure 3). Our data demonstrate that *Ythdf1* deletion in DCs sensitized tumors to IR in murine colon cancer and melanoma.

### Ythdf1 deficiency increases the cross-priming capacity of DCs in the context of IR.

**Figure 2:**
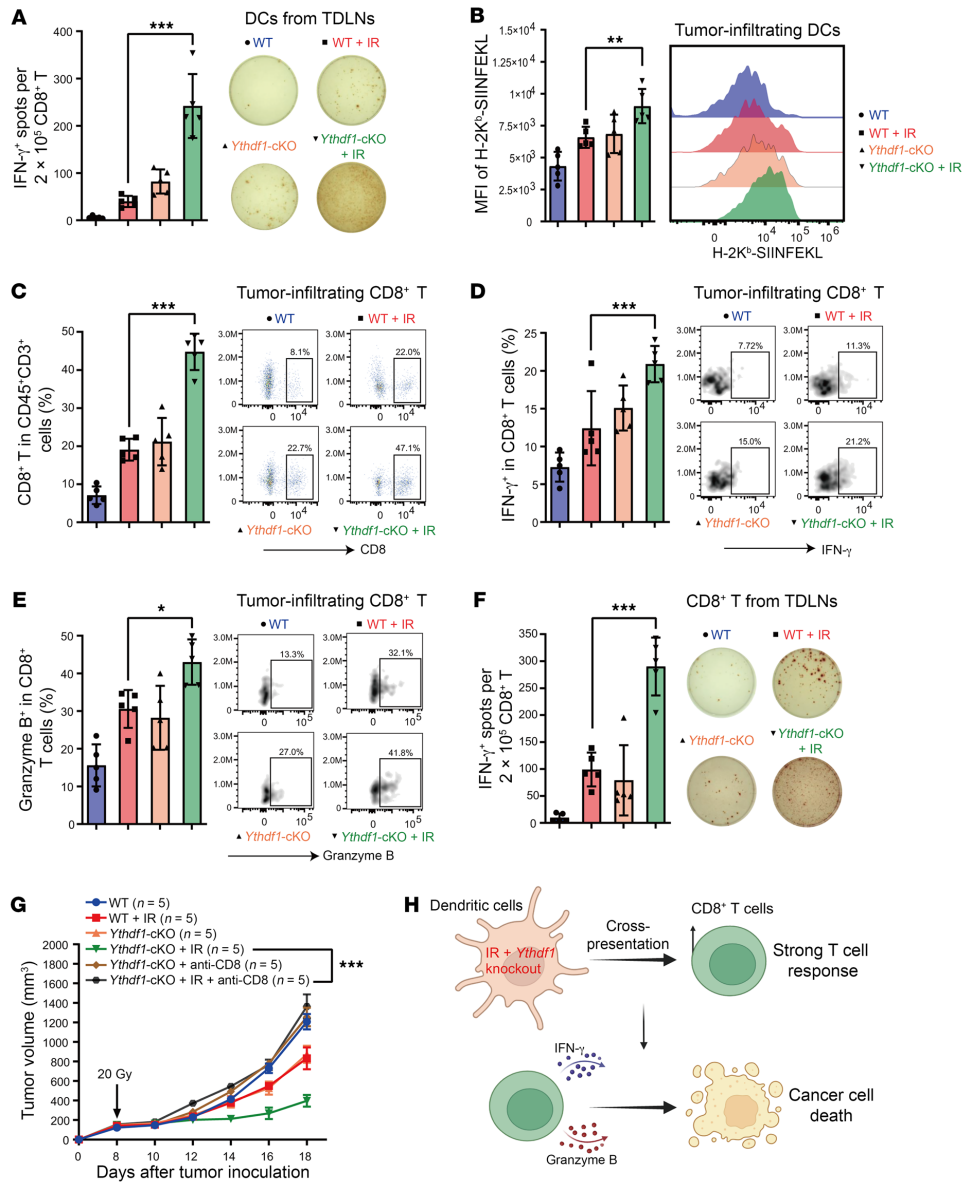
*Ythdf1* deficiency increases the cross-priming capacity of DCs in the context of IR. (**A** and **B**) WT and *Ythdf1*-cKO mice were injected s.c. with B16-OZ cells. Tumor-bearing mice were treated with local IR (20 Gy, 1 dose) when tumor volumes reached 100–200 mm^3^. On day 5 after IR, CD11c^+^ cells from TDLNs were isolated and cocultured with OT-I T cells for 3 days, and then IFN-γ–producing cells were enumerated by ELISPOT (*n* = 5) (**A**); in tumor-infiltrating DCs (CD45^+^F4/80^–^CD11c^+^MHC-II^+^), the formation of H-2K^b^-SIINFEKL was detected by flow cytometry (*n* = 5) (**B**). (**C**–**E**). On day 8 after IR, the proportions of CD8^+^ T cells (CD45^+^CD3^+^CD8^+^) (**C**), IFN-γ (**D**), and granzyme B (**E**) in CD8^+^ T cells were detected by flow cytometry (*n* = 5). (**F**) On day 8 after IR, CD8^+^ T cells were isolated from TDLNs. Tumor antigen–specific CD8^+^ T cell function was measured via ELISPOT by coculturing CD8^+^ T cells with 5 μg/mL OT-I peptide (*n* = 5). (**G**) A dose of 200 μg anti-CD8 mAb was delivered twice weekly by i.p. injection to deplete CD8^+^ T cells, starting 1 day before IR. Tumor growth was monitored after IR. (**H**) Proposed model of how *Ythdf1* KO in DCs sensitizes a tumor to IR by increasing the antitumor activity of CD8^+^ T cells. Data are presented as the mean ± SEM. Data are representative of 2 or 3 independent experiments. One-way ANOVA with Tukey’s multiple-comparison test (**A**–**F**) and 2-way ANOVA with Tukey’s multiple-comparison test (**G**). *P < 0.05, **P < 0.01, and ***P < 0.001.

To assess whether distinct DC subpopulations are implicated in antitumor effects of IR in *Ythdf1-*cKO mice, we profiled the B16-OZ tumor–infiltrating DCs by flow cytometry. The percentage of XCR1^+^ (DC1) and CD11b^+^ (DC2) DCs did not change when comparing irradiated and nonirradiated WT and *Ythdf1*-cKO mice (Supplemental Figure 4, A and B). We thus reasoned that *Ythdf1* deficiency may influence DC function. To test this, we examined the cross-presentation capacity of DCs by performing an IFN-γ–based ELISPOT assay using DCs isolated from tumor-draining lymph nodes (TDLNs) of B16-OZ tumor–bearing mice. The results showed that DCs sorted from *Ythdf1*-cKO mice subjected to IR (*Ythdf1*-cKO+IR mice) demonstrated a 5-fold increase in cross-priming ability compared with those from WT+IR mice (*P* < 0.001, Figure 2A). Moreover, we analyzed the frequency of tumor-infiltrating DCs (CD45^+^F4/80^–^CD11c^+^MHC-II^+^) expressing MHC-I–SIINFEKL and determined that IR treatment resulted in a significant increase in the level of MHC-I–SIINFEKL complexes in DCs from *Ythdf1*-cKO+IR mice compared with WT+IR mice (*P* < 0.01, Figure 2B). In addition, we observed no changes in CD80 or CD86 staining and no changes in the phagocytosis of tumor-infiltrating DCs in tumors (detected by the expression of zsGreen), ruling out the effects of IR or *Ythdf1* deletion on the costimulatory molecules or phagocytic activity in *Ythdf1*-cKO mice following IR (Supplemental Figure 4, C–E). Similar results were observed in *Ythdf1*-KO mice that underwent IR treatment (Supplemental Figure 4, A–E, and Supplemental Figure 5, A and B). Our data demonstrate that *Ythdf1* deletion enhanced the cross-presentation capacity of DCs in the context of IR.

To assess whether the antitumor effects of IR in *Ythdf1*-cKO mice depend on CD8^+^ T cells, we examined B16-OZ tumor–infiltrating T cells by flow cytometry. Flow data showed a significant increase in the number of total CD8^+^ T cells, IFN-γ^+^CD8^+^ T cells, and granzyme B^+^CD8^+^ T cells in *Ythdf1*-cKO+IR mice compared with WT+IR mice (*P* < 0.001, *P* < 0.001, and *P* < 0.05, respectively, Figure 2, C–E). These results indicate that *Ythdf1* deletion in DCs enhanced IR-induced CD8^+^ T cell cytotoxicity. To further investigate changes in CD8^+^ T cell priming, we isolated CD8^+^ T cells from TDLNs of B16-OZ tumor–bearing mice and treated the cells with the MHC-I–restricted peptide SIINFEKL of OVA. An IFN-γ–based ELISPOT assay showed that CD8^+^ T cells isolated from *Ythdf1*-cKO+IR tumor–bearing mice had a greater number of IFN-γ^+^ spots than did those isolated from WT+IR mice (Figure 2F). This result indicates that *Ythdf1* deletion in DCs enhanced IR-induced T cell priming against tumor-specific antigens. We also observed similar results in *Ythdf1*-KO mice (Supplemental Figure 5, C–F). In addition, CD8^+^ T cell depletion using an anti-CD8α antibody completely abrogated the antitumor effect of IR in the *Ythdf1*-cKO groups (Figure 2G). These results demonstrate that CD8^+^ T cells are essential for the enhanced antitumor effect of IR in *Ythdf1-*cKO mice (Figure 2H).

### IR increases YTHDF1 expression in DCs via STING/IFN-I signaling.

**Figure 3:**
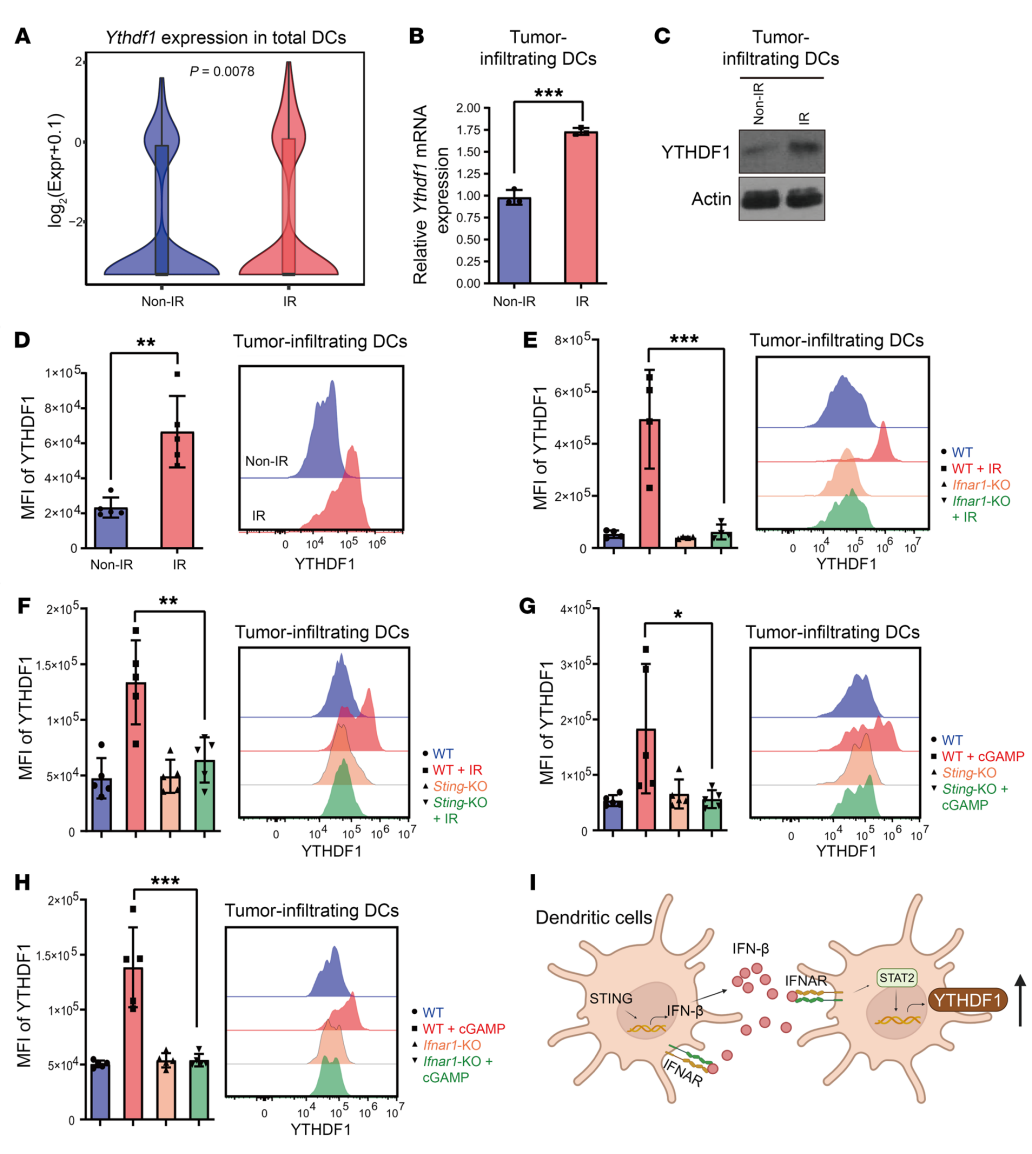
IR increases YTHDF1 expression in DCs via STING/IFN-I signaling. (**A**) Violin plot showing the expression of *Ythdf1* in total DCs from the scRNA-Seq data collected from tumor-infiltrating CD45^+^ cells of MC38-bearing mice on day 4 after IR. (**B**–**D**). WT mice were injected s.c. with B16-OZ cells. Tumor-bearing mice received local IR (20 Gy, 1 dose) when the tumor volume reached 100–200 mm^3^. Tumor-infiltrating DCs (CD45^+^F4/80^–^CD11c^+^MHC-II^+^) were sorted for detection of the expression of YTHDF1 on day 5 after IR via qPCR (*n* = 3) (**B**) and Western blotting (**C**). YTHDF1 expression in tumor-infiltrating DCs by intracellular staining and flow cytometry (*n* = 5) (**D**). (**E** and **F**) B16-OZ tumor–bearing *Ifnar1*-KO mice (**E**) and *Sting-*KO mice (**F**) received local IR (20 Gy, 1 dose), and the expression of YTHDF1 in tumor-infiltrating DCs was detected via flow cytometry on day 1 after IR (*n* = 4 or 5). (**G** and **H**) B16-OZ tumor–bearing *Sting-*KO mice (**G**) and *Ifnar1*-KO mice (**H**) were treated with 2 doses of 10 μg 2′3′-cGAMP (i.t.), and the expression of YTHDF1 of tumor-infiltrating DCs was detected by flow cytometry (*n* = 5). (**I**) Proposed mechanism of how IR induces YTHDF1 expression in DCs. IR-induced STING/IFN-I signaling increases the expression of YTHDF1 in DCs. Data are presented as the mean ± SEM. Data are representative of 2 or 3 independent experiments (**B**–**H**) Wilcoxon Mann-Whitney *U* test (**A**), 2-sided, unpaired Student’s *t* test (**B** and **D**), and 1-way ANOVA with Tukey’s multiple-comparison test (**E**–**H**). *P < 0.05, **P < 0.01, and ***P < 0.001.

To investigate the role of RT-induced YTHDF1 expression in murine tumors models, we analyzed our single-cell RNA-Seq data on CD45^+^ immune cells isolated from nonirradiated and irradiated MC38 tumors (32). We constructed a violin plot analysis to score for *Ythdf1* expression and noted a significant increase in the expression of *Ythdf1* in total DCs (*P* = 0.0078, Figure 3A). We verified that IR indeed increased YTHDF1 expression in tumor-infiltrating DCs at both mRNA and protein levels (Figure 3, B–D). Increased YTHDF1 expression was observed as early as 24 hours after IR treatment (Supplemental Figure 6A). Interestingly, fractionated radiotherapy had similar effects on YTHDF1 expression as a single high-dose IR treatment (Supplemental Figure 6B). Moreover, an ex vivo assay with cocultured bone marrow–derived DCs (BMDCs) and B16-OZ cells showed that irradiated tumor cells had increased YTHDF1 expression at both mRNA and protein levels (Supplemental Figure 6, C–E). These results demonstrate that IR induced YTHDF1 in DCs in both clinical and preclinical settings.

We then sought to determine how IR upregulates YTHDF1 expression in DCs. Our exploratory analyses using the TIMER database revealed a strong positive correlation between IFN-α/β receptor (IFNAR) and YTHDF1 in 4 cancer types including melanoma, glioma, prostate, and liver cancer (*P* < 0.001, Supplemental Figure 6, F and G). Given that irradiated tumor cells induce IFN-I production in DCs (18, 21), we proposed that IFN-I signaling could be involved in the IR-induced increase in YTHDF1 expression in DCs. By analyzing tumor-infiltrating DCs grown in WT or *Ifnar1*-KO mice, we demonstrated that IFNAR deficiency completely abolished IR induction of YTHDF1 expression observed in tumors of WT animals (Figure 3E). YTHDF1 expression in BMDCs from *Ifnar1-*KO mice was not increased by irradiated tumor cells (Supplemental Figure 6H). Moreover, we found that IFN-β1 directly increased YTHDF1 expression in BMDCs (Supplemental Figure 6, I and J). Taken together, our data demonstrate that IFN-I signaling is necessary and probably sufficient for YTHDF1 induction in DCs in the context of IR.

We next sought to determine which downstream factors regulated by IFN-I are required for YTHDF1 expression in DCs. Data from a public dataset of ChIP-Seq conducted in mouse bone marrow–derived macrophages indicate that STAT2 can directly bind to the *Ythdf1* promoter region (Supplemental Figure 6K). STAT2 has been reported as a key transcriptional factor in IFN-I signaling (33, 34). Our ChIP–quantitative PCR (qPCR) results confirmed that STAT2 directly bound to the *Ythdf1* promoter region (–500 kb ~ –1,000 kb) (Supplemental Figure 6L). Moreover, STAT2 knockdown decreased the expression of *Ythdf1* and abolished the IR-induction of *Ythdf1* in DCs (Supplemental Figure 6, M–O). Our results suggest that IR induced YTHDF1 expression via the IFN-I/STAT2 axis.

Irradiated tumor cells can activate STING signaling in DCs and consequent IFN-I production (18, 21). We therefore speculated that STING signaling could be involved in IR–IFN-I–mediated YTHDF1 induction. To test this, we measured YTHDF1 expression in tumor-infiltrating DCs from *Sting*-KO mice and found that IR did not significantly induce YTHDF1 expression in DCs from *Sting*-KO mice compared with DCs from WT mice (Figure 3F and Supplemental Figure 6P), suggesting that *Sting* KO abolished IR-induced YTHDF1 in DCs. Moreover, our results showed that intratumoral (i.t.) treatment of 2′3′-cGAMP (STING agonist) increased the expression of YTHDF1 in tumor-infiltrating DCs in WT mice but not in *Sting*-KO mice (Figure 3G). We found that 2′3′-cGAMP upregulated YTHDF1 expression in BMDCs in vitro (Supplemental Figure 6, Q and R). These results indicate that STING signaling was required to mediate IR induction of YTHDF1 expression.

To determine whether IFN-I signaling is required for STING-mediated YTHDF1 induction, we used a STING agonist (2′3′-cGAMP) to mimic the IR stress that activates STING signaling. Our flow results showed that YTHDF1 expression in DCs from *Ifnar1-*KO mice was not increased by 2′3′-cGAMP (Figure 3H and Supplemental Figure 6S), indicating that STING-induced YTHDF1 expression relied on IFN-I. Collectively, our data demonstrate that IR upregulated YTHDF1 expression through STING/IFN-I signaling (Figure 3I).

### Ythdf1 deletion enhances STING/IFN-I signaling in DCs.

**Figure 4:**
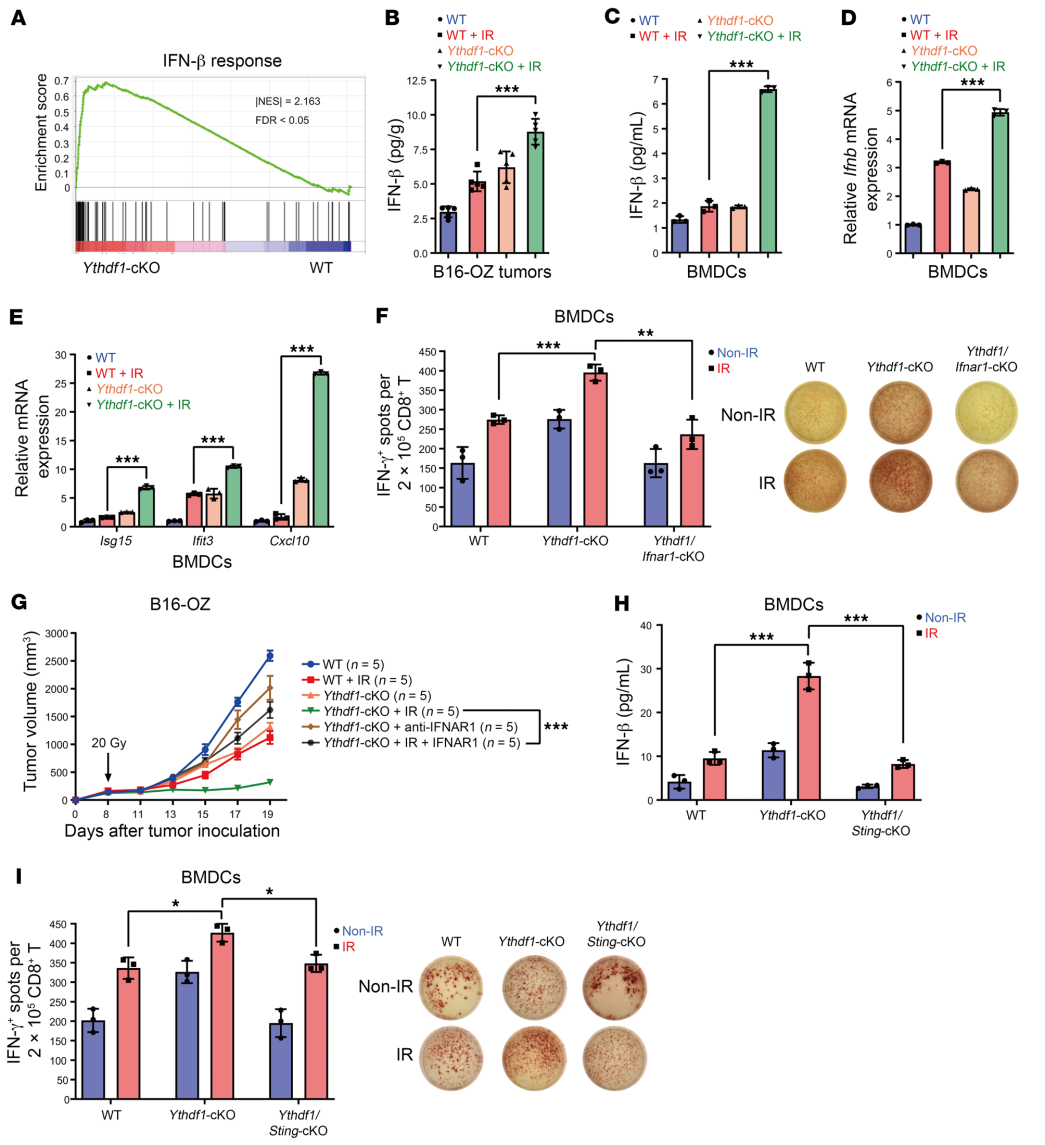
*Ythdf1* deletion enhances STING/IFN-I signaling in DCs. (**A**) GSEA showing an increased IFN-β response in DCs with *Ythdf1* deletion versus WT DCs. (**B**) WT and *Ythdf1*-cKO mice were injected s.c. with B16-OZ cells. Tumor-bearing mice underwent local IR (20 Gy, 1 dose) when the tumor volume reached 100–200 mm^3^, and tumors were excised on day 3 after IR for IFN-β ELISA (*n* = 5). (**C**–**E**) BMDCs from *Ythdf1*-cKO mice were cocultured with 40 Gy–pretreated or nonirradiated B16-OZ cells for 8 hours. Purified CD11c^+^ cells were incubated for another 2 days. Supernatants were collected to measure IFN-β by ELISA (*n* = 3) (**C**). Purified CD11c^+^ cells were collected to measure mRNA levels of *Ifnb* (**D**) and *Isg15, Ifit3*, and *Cxcl10* (**E**) by qPCR (*n* = 3). (**F**) BMDCs from *Ythdf1*-cKO and *Ythdf1/Ifnar1*-cKO mice were cocultured with B16-OZ cells for 8 hours. Purified CD11c^+^ cells were incubated with CD8^+^ T cells from OT-I mice for another 3 days. IFN-γ–producing cells were enumerated by ELISPOT (*n* = 3). (**G**) B16-OZ tumors in WT and *Ythdf1*-cKO mice were treated with IR and/or 200 μg anti-IFNAR1 mAb injected i.t. twice weekly. Tumor growth was monitored after IR. (**H** and **I**) BMDCs from *Ythdf1*-cKO and *Ythdf1*/*Sting*-cKO mice were cocultured with B16-OZ cells for 8 hours. Purified CD11c^+^ cells were incubated for an additional 2 days. IFN-β levels in supernatants were measured by ELISA (*n* = 3) (**H**). Purified CD11c^+^ cells were incubated with CD8^+^ T cells from OT-I mice for another 3 days. Then IFN-γ–producing cells were enumerated by ELISPOT (*n* = 3) (**I**). Data are presented as the mean ± SEM and are representative of 2 or 3 independent experiments. (**B**–**I**) One-way ANOVA with Tukey’s multiple-comparison test (**B**–**F**, **H**, and **I**) and 2-way ANOVA with Tukey’s multiple-comparison test (**G**). *P < 0.05, **P < 0.01, and ***P < 0.001.

To investigate the mechanisms governing YTHDF1 function in DCs in the context of IR, we analyzed our RNA-Seq data from BMDCs derived from WT and *Ythdf1-*KO mice. The gene set enrichment analysis (GSEA) showed that “IFN-I response” signaling was enriched in *Ythdf1-*KO DCs, but not in WT DCs (Figure 4A). Considering our previous results indicating that IR-induced IFN-I production in DCs mediates their cross-priming ability and the antitumor effects of IR (21, 23), we hypothesized that *Ythdf1* deletion could be associated with the enhanced IR-induced IFN-I production. To test this, we first measured the production of IFN-β in tumor tissues by ELISA. We observed significantly greater IFN-β production in B16-OZ tumors from *Ythdf1*-cKO+IR mice compared with WT+IR mice (>1.5-fold, *P* < 0.001, Figure 4B). Moreover, compared with WT-BMDCs, *Ythdf1*-cKO BMDCs exhibited a significantly higher levels of IFN-β production when cocultured with irradiated tumor cells (*P* < 0.001, Figure 4, C and D). *Ythdf1*-cKO BMDCs demonstrated increased gene expression of IFN-stimulated genes (ISGs) such as *Isg15*, *Ifit3*, and *Cxcl10* (35, 36) compared with those expression in WT BMDCs (Figure 4E). Our results indicate that *Ythdf1* deletion enhanced IR-triggered IFN-I production in DCs.

To determine whether IFN-I signaling is required for the enhanced cross-priming activity of DCs and antitumor effects in *Ythdf1*-cKO mice with IR treatment, we used *Cd11c^Cre^* *Ythdf1^fl/fl^* and *Ifnar1^fl/fl^* conditional double-KO mice for ex vivo and in vivo experiments. We found that *Ythdf1*-cKO BMDCs induced a greater cross-priming capacity when cocultured with irradiated B16-OZ cells compared with WT BMDCs; however, this increase was abrogated when *Ifnar1* was knocked out in *Ythdf1*-cKO BMDCs (*Ythdf1*/*Ifnar1*-cKO, Figure 4F). Moreover, blocking IFN-I signaling by IFNAR1 neutralizing antibody also abrogated the antitumor efficacy of IR in *Ythdf1*-cKO mice (Figure 4G). These results indicate that IFN-I signaling is critical for the enhanced antitumor efficacy of IR in *Ythdf1*-cKO mice. According to our previous findings that STING signaling is a key pathway underlying IFN-I production in DCs (18, 21), we proposed that the STING pathway is involved in IFN-β production induced by *Ythdf1* deletion. To test this, we generated *Ythdf1* and *Sting* double-cKO mice (*Ythdf1*/*Sting*-cKO) and isolated BMDCs to measure IFN-β expression and BMDC cross-presentation activity. As expected, we observed a significant decrease in IFN-β production and cross-presentation function (based on an IFN-γ ELISPOT assay) in *Ythdf1*/*Sting*-cKO BMDCs compared with *Ythdf1*-cKO BMDCs in the context of IR (*P* < 0.001, *P* < 0.05 respectively, Figure 4, H and I). Collectively, our results indicate that *Ythdf1* deletion in turn activated STING signaling and thereby promoted IFN-β production in DCs. These results also demonstrate that YTHDF1 dampened DC function by inhibiting or abolishing STING/IFN-I signaling.

### Ythdf1 deletion diminishes the cathepsin-mediated decrease in STING expression in DCs.

**Figure 5:**
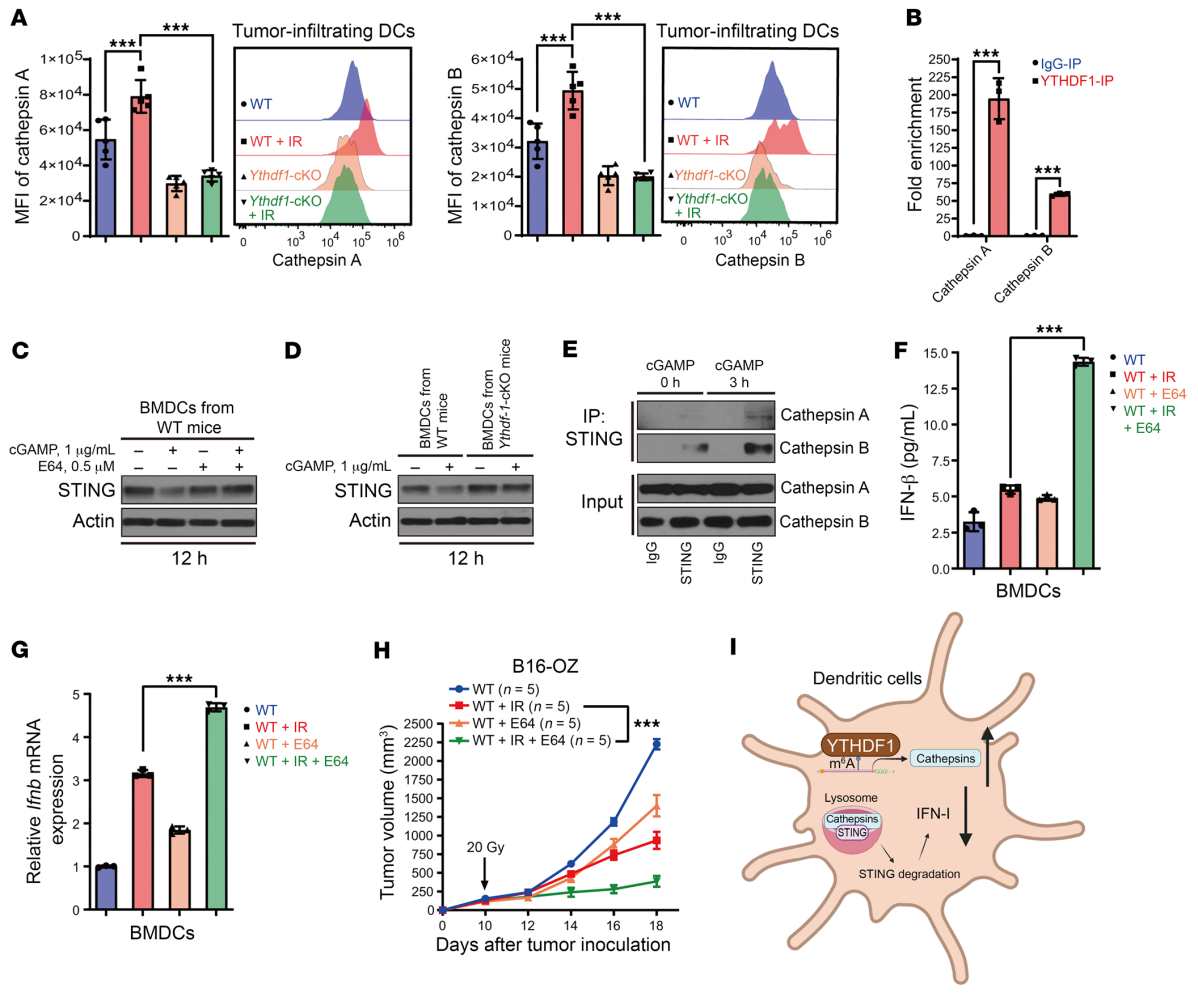
*Ythdf1* deletion diminishes the cathepsin-mediated decrease in STING expression in DCs. (**A**) WT and *Ythdf1*-cKO mice were injected s.c. with B16-OZ cells. Tumor-bearing mice were treated with local IR (20 Gy, 1 dose) when the tumor volume reached 100–200 mm^3^. Expression of cathepsins A and B in tumor-infiltrating DCs (CD45^+^F4/80^–^CD11c^+^MHC-II^+^) was detected via flow cytometry on day 5 after IR (*n* = 5). (**B**) BMDCs from WT mice were cocultured with 40 Gy–pretreated B16-OZ cells for 24 hours. Purified CD11c^+^ cells were collected to measure the enrichment of cathepsin A and B mRNA in the YTHDF1-immunoprecipitated RNA fraction (*n* = 3). (**C**) BMDCs from WT mice were treated with 2′3′-cGAMP and E64 for 12 hours, and STING expression was detected by Western blotting. (**D**) WT BMDCs and BMDCs with *Ythdf1* deletion were treated with 2′3′-cGAMP for 12 hours, and STING expression was detected by Western blotting. (**E**) BMDCs from WT mice were stimulated with 2′3′-cGAMP. After immunoprecipitation with STING antibody, the expression of cathepsins A and B in whole-cell lysates was detected by Western blot. (**F** and **G**) BMDCs from WT mice were pretreated with E64 for 24 hours and then cocultured with 40 Gy–pretreated or nonirradiated B16-OZ cells for 8 hours. Purified CD11c^+^ cells were incubated for another 2 days. Supernatants were collected to measure IFN-β by ELISA (*n* = 3) (**F**), and cells were collected to measure *Ifnb* mRNA levels (*n* = 3) (**G**). (**H**) B16-OZ tumor–bearing WT mice were treated with local IR (20 Gy, 1 dose) and 50 μM E64 (i.t., daily). Tumor growth was monitored after IR. (**I**) Mechanism of how YTHDF1 affects STING/IFN-I signaling in DCs. IR-induced YTHDF1 increases the degradation of STING via cathepsins in the lysosomes of DCs, ultimately leading to decreased IFN-I production. Data are represented as the mean ± SEM and are representative of 2 or 3 independent experiments. Two-sided, unpaired Student’s *t* test (**B**), 1-way ANOVA with Tukey’s multiple-comparison test (**A**, **F**, and **G**), and 2-way ANOVA with Tukey’s multiple-comparison test (**H**). ***P < 0.001.

We next sought to investigate mechanisms by which *Ythdf1* deletion enhances STING/IFN-I pathway activation in DCs. Lysosomal cathepsins are the targets of YTHDF1 in DCs and are decreased in DCs with *Ythdf1* deletion (10). We reasoned that *Ythdf1* deletion could contribute to the reduced cathepsins in IR-treated DCs, as IR increased the expression of YTHDF1 in DCs. Our results demonstrated that IR increased the expression of cathepsins A and B in B16-OZ tumor–infiltrating DCs, which was abolished in *Ythdf1*-deleted DCs (Figure 5A). Moreover, RNA immunoprecipitation–qPCR (RIP-qPCR) analysis determined that YTHDF1 bound to cathepsins A and B in DCs in the context of IR (Figure 5B). These findings suggest that cathepsins are primary targets of YTHDF1 in DCs under IR conditions.

STING protein is translocated and degraded in lysosomes when STING signaling is activated (37, 38). Considering the close relationship between YTHDF1 and lysosome function in DCs and that cathepsins are the primary proteases in lysosomes (10, 39), we hypothesized that YTHDF1 negatively regulates the expression of IFN-I in DCs via cathepsin-mediated STING degradation. To test this, we first measured total STING levels in 2′3′-cGAMP–activated BMDCs and found that STING protein levels decreased several hours after being activated by 2′3′-cGAMP (Supplemental Figure 7), which is consistent with previous studies showing that STING protein is degraded after DNA stimulation in mouse embryonic fibroblasts (38). The Western blot assay results confirmed that protein levels of STING were restored by treatment with the cathepsin inhibitor E64 or *Ythdf1* KO in DCs (Figure 5, C and D). Moreover, a co-IP assay using an anti-STING antibody showed that STING directly bound to cathepsins A and B after cGAMP stimulation (Figure 5E). Furthermore, IR induced greater IFN-β production in DCs treated with E64 (Figure 5, F and G). For the in vivo antitumor effects, E64 treatment markedly improved the response to IR in WT mice (Figure 5H), similar to what was observed in *Ythdf1-*cKO mice (as shown in Figure 1). These results suggest that *Ythdf1* deletion in DCs decreased cathepsin A and B levels and prevented STING degradation. *Ythdf1* deficiency thereby promoted IFN-I production and DC function in the context of IR (Figure 5I).

### DC vaccines with YTHDF1 deletion/inhibition improve the response to RT and immunotherapy in murine cancers.

**Figure 6:**
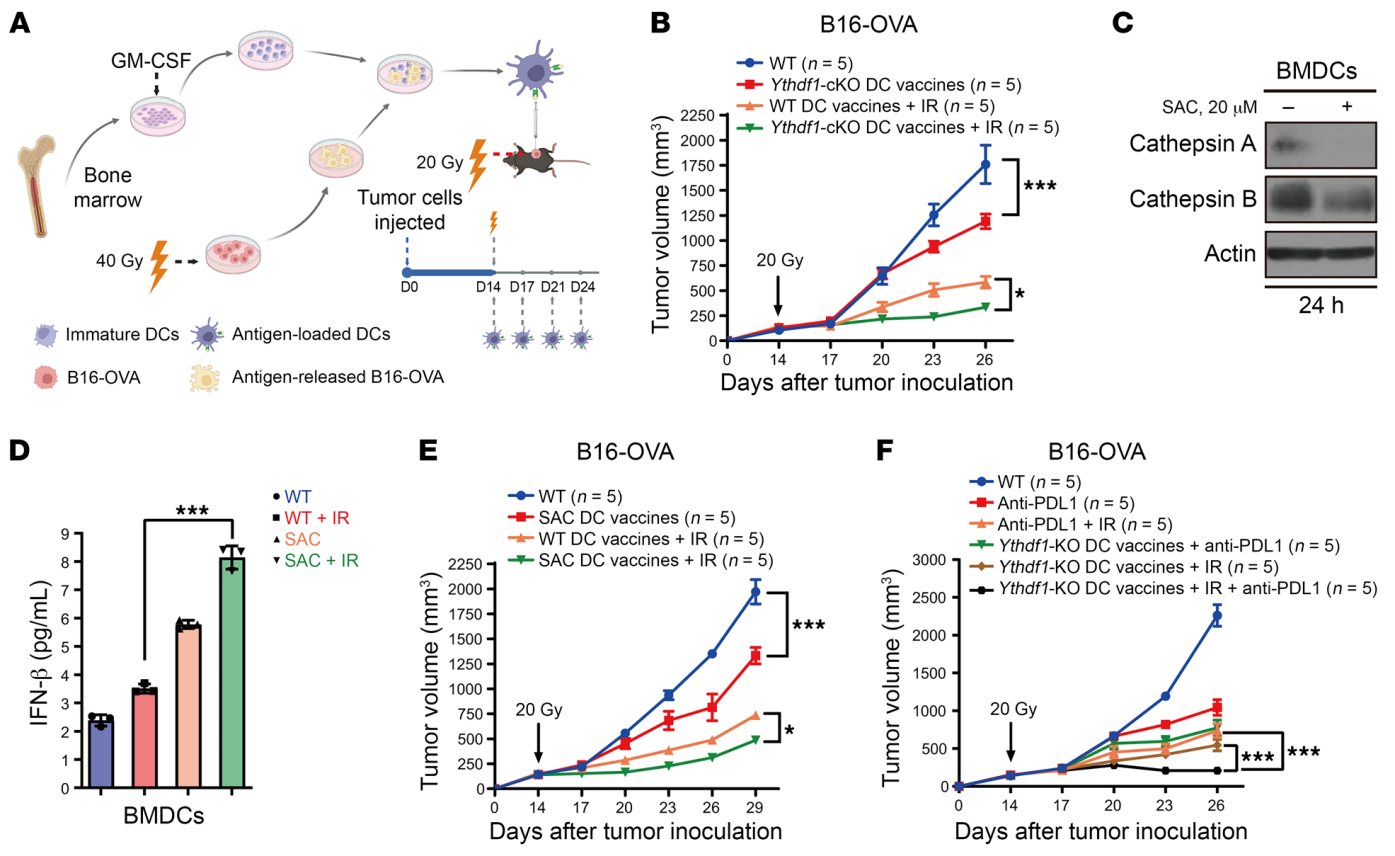
DC vaccines with YTHDF1 deletion/inhibition improve the response to RT and immunotherapy in murine cancers. (**A**) Schematic diagram of the DC vaccine treatment plan. D, day. (**B**) WT mice were injected s.c. with B16-OVA cells. When the tumor volume reached 100–200 mm^3^, tumor-bearing mice were treated with IR (20 Gy, 1 dose) and antigen-pulsed BMDCs from WT or *Ythdf1*-KO mice (twice weekly by i.t. injection). (**C**) BMDCs from WT mice were treated with 20 μM SAC for 24 hours, and the expression of cathepsins A and B was detected by Western blotting. (**D**) BMDCs from WT mice were pretreated with SAC for 24 hours and then cocultured with 40 Gy–pretreated or nonirradiated B16-OZ cells for 8 hours. Purified CD11c^+^ cells were incubated for another 2 days, and the supernatants were collected to measure IFN-β (*n* = 3). (**E**) B16-OVA tumor–bearing mice were treated with IR (20 Gy, 1 dose) and antigen-pulsed BMDCs from WT mice with or without SAC treatment (twice weekly by i.t. injection). (**F**) B16-OVA tumor–bearing mice were treated with IR (20 Gy, 1 dose), antigen-pulsed BMDCs from *Ythdf1*-KO mice (twice weekly by i.t. injection), and anti–PD-L1 (twice weekly by i.p. injection). Data are represented as the mean ± SEM and are representative of 2 or 3 independent experiments. One-way ANOVA with Tukey’s multiple-comparison test (**D**) and 2-way ANOVA with Tukey’s multiple-comparison test (**B**, **E**, and **F**). *P < 0.05 and ***P < 0.001.

Our results demonstrated that when YTHDF1 was depleted, both the cross-presentation and priming function of DCs were significantly enhanced, which led to superior tumor control in the context of IR. To investigate the potential clinical translation, we generated prototype DC vaccines using *Ythdf1*-deficient DCs (Figure 6A). The transfer of DC vaccines generated from *Ythdf1*-deficient DCs (*Ythdf1*-KO DC vaccines) significantly enhanced the antitumor efficacy of IR compared with DC vaccines generated from WT DCs (WT DC vaccines) (*P* < 0.05, Figure 6B), suggesting the potential beneficial clinical applications of combining *Ythdf1*-KO DC vaccines and RT. To establish proof of principle for clinical translation and utility, we generated a prototype DC vaccine using BMDCs treated with a known YTHDF1 inhibitor, salvianolic acid C (SAC) (SAC DC vaccines) (40). We verified that SAC treatment decreased the expression of cathepsins A and B and increased IR-induced IFN-I production (Figure 6, C and D) in a manner similar to the results obtained with *Ythdf1-*deficient DCs. More important, SAC DC vaccines resulted in significantly better IR-induced antitumor efficacy compared with WT DC vaccines (*P* < 0.05, Figure 6E), suggesting that SAC DC vaccines can sensitize tumors to IR at a level similar to that seen with *Ythdf1*-KO vaccines.

It has been reported that IFNs increase the expression of PD-L1 (41–43). If *Ythdf1*-cKO+IR mice were to produce higher levels of IFN-β and IFN-γ compared with controls, we would expect increased PD-L1 expression in tumor-infiltrating DCs. Indeed, using flow cytometry, we observed a higher level of PD-L1 in tumor-infiltrating DCs from *Ythdf1*-cKO+IR mice compared with those from WT mice (Supplemental Figure 8). These results suggest that PD-L1 blockade could enhance the antitumor effect of IR in *Ythdf1*-KO DC vaccine–treated mice. Indeed, the IR and anti–PD-L1 combination resulted in almost complete tumor regression in mice treated with *Ythdf1*-KO DC vaccines (Figure 6F). These results also showed that *Ythdf1*-KO DC vaccines significantly enhanced the tumor control of IR+anti-PD-L1 combination treatment (*P* < 0.001, Figure 6F).

## Discussion

Here, we report both clinical and preclinical findings: YTHDF1 elevation in DCs after RT was associated with unfavorable outcomes in patients, and IR-induced YTHDF1 in DCs impaired the antitumor immunity elicited by IR in multiple murine cancer models. Deletion of YTHDF1 in DCs improved the therapeutic effect of RT and radioimmunotherapy by increasing the cross-priming capacity of DCs. We found that IR-induced STING/IFN-I signaling could upregulate YTHDF1 expression; the elevated YTHDF1 levels in turn triggered STING degradation by increasing lysosomal cathepsin levels, and subsequently, the reduction in STING impaired IFN-I production and the cross-priming capacity of DCs. This IR/YTHDF1/STING/IFN-I circuit in DCs represents what we believe to be a previously unrecognized pathway contributing to extrinsic radioresistance. Our study delineates a deeper link between YTHDF1 and IFN-I–mediated antigen presentation in DCs in response to IR and reveals YTHDF1/m^6^A as a new layer of STING suppression in the immune system.

IR induces antitumor immunity and immunosuppressive effects in DCs, and blocking these immunosuppressive effects enhances the antitumor effects (18, 19, 44–46). On the basis of our clinical and preclinical data, we posit that YTHDF1 acts as a crucial immune-suppressive factor/mechanism or innate checkpoint in DCs following IR and has potential as a promising therapeutic target to enhance antitumor efficacy. Considering the broad expression profile of YTHDF1 in various immune cells, we cannot rule out the possibility that YTHDF1 in other types of cells may also contribute to extrinsic or intrinsic radioresistance. Therefore, future elucidation of the roles of YTHDF1 in various immune and cancer cells will be important in the context of the antitumor effects of IR.

We present what we believe to be a previously unknown mechanism of STING degradation following IR-induced YTHDF1 expression; this degradation likely occurs in lysosomes by cathepsins, which are the targets of the IR-induced YTHDF1. Our results provide evidence that YTHDF1 acts as a negative regulator of STING/IFN-I signaling in DCs. Targeting YTHDF1 could enhance the antitumor efficacy of IR via restoration of STING signaling. It is noteworthy that reduced cathepsin levels may also promote antigen presentation, and, beyond cathepsins, YTHDF1 may interact with additional targets under different conditions to exert its intricate regulatory effects upon the STING/IFN-I signaling pathway, a question necessitating further investigation.

Clinical results have indicated that combining DC vaccines with RT is a promising approach (47, 48). We report here that experimental prototype DC vaccines using *Ythdf1-*deficient DCs or with YTHDF1 inhibitor treatment enhanced the antitumor effects of IR more effectively than did DC vaccines from WT or untreated DCs. Use of a small-molecule YTHDF1 inhibitor to prepare DC vaccines shows great promise for clinical translation. Such an approach would preclude the need for genetic manipulation of human DCs, and it has the potential to simplify the process of vaccine preparation. Further support for the efficacy of our DC vaccines will require additional preclinical and clinical data. Use of immune checkpoint inhibitors in the clinic is now prevalent due significant research advancements in recent years in the field of antitumor immunotherapy (49, 50). We have observed that IR and *Ythdf1* deletion upregulated the expression of PD-L1 in DCs. *Ythdf1*-deficient DC vaccines enhanced the antitumor effects of IR+anti-PD-L1, suggesting that triple therapy is worth further investigation in clinical research to determine its therapeutic efficacy.

## Methods

### Sex as a biological variable.

Both sexes were used for human and mouse studies. Sex was not considered as a biological variable.

### Mice.

All C57BL/6 mice were purchased from Envigo. *Cd11c*^cre^, *Sting* whole-body KO (*Sting*-KO), *Ifnar1* whole-body KO (*Ifnar1*-KO), and *Ifnar1^fl/fl^* and OT-I transgenic mice were purchased from The Jackson Laboratory. *Sting^fl/fl^* mice were provided by John Cambier (National Jewish Health, Denver, Colorado, USA). *Ythdf1^fl/fl^* mice were generated by Bin Shen’s Laboratory (Nanjing Medical University, Nanjing, Jiangsu, China). Briefly, the *Ythdf1*-KO first embryonic stem cell clone was purchased from the CAM-SU Genomic Resource Center and was microinjected into mouse blastocysts to generate founder mice. These mice were crossed with flipper mice to excise the FRT-flanked selection. *Ythdf1* whole-body KO (*Ythdf1*-KO) mice were generated as previously described (3). DC-specific *Ythdf1*, *Sting*, and *Ifnar1* KO mice were generated by crossing *Ythdf1^fl/fl^*, *Sting^fl/fl^*, and *Ifnar1^fl/fl^* mice with *Cd11c^Cre^* mice, respectively (*Ythdf1*-cKO, *Sting*-cKO and *Ifnar1*-cKO). DC-specific *Ythdf1/Sting and Ythdf1/Ifnar1* double-KO mice were generated by crossing *Sting*-cKO and *Ifnar1*-cKO mice with *Ythdf1*-cKO mice, respectively (*Ythdf1/Sting*-cKO, *Ythdf1/Ifnar1*-cKO). Mice at 6–12 weeks of age were maintained under specific pathogen–free conditions.

### Primary cell cultures.

Single-cell suspensions of bone marrow cells were obtained from C57BL/6J, *Ythdf1*-cKO, *Ifnar1*-KO, *Sting*-KO, *Ythdf1/Ifnar1*-cKO, and *Ythdf1/Sting*-cKO mice. The cells were cultured in IMDM (Gibco, Thermo Fisher Scientific, catalog 12440046) containing 10% FBS (GeminiBio, catalog 100106500), 1% penicillin-streptomycin solution (Gibco, Thermo Fisher Scientific, catalog 15140122), 1% sodium pyruvate (Gibco, Thermo Fisher Scientific, catalog 11360070), 1% MEM nonessential amino acids (Gibco, Thermo Fisher Scientific, catalog 11140050), 55 mM β-mercaptoethanol (Gibco, Thermo Fisher Scientific, catalog 21985023), and 100 ng/mL human FLT3L (BioLegend, catalog 550606) for 9 days. Fresh media with FLT3L were added to the culture on day 5, and BMDCs were obtained on day 9 (51–53). All cells were maintained in a humidified incubator at 37°C and 5% CO_2_.

### Cell lines.

MC38 and B16 cells were purchased from the American Type Culture Collection (ATCC). B16-OVA and B16-OZ were preserved in our laboratory. These cancer cells were grown in DMEM (Gibco, Thermo Fisher Scientific, catalog 11965092) supplemented with 10% FBS (GeminiBio, catalog 100106500) and 1% penicillin-streptomycin solution (Gibco, Thermo Fisher Scientific, catalog 15140122). All cells were maintained in a humidified incubator at 37°C and 5% CO_2_.

### Patient samples.

PBMCs were obtained from patients with metastatic NSCLC enrolled in a clinical trial at our institution (COSINR study, NCT03223155) (31). The patients were treated with sequential or concurrent SBRT and immune checkpoint blockade therapy (ipilimumab and nivolumab). PBMCs were collected prior to treatment and following completion of SBRT.

### Tumor growth and treatments.

MC38, B16-OZ, or B16-OVA tumor cells (1 × 10^6^) were s.c. injected into the right flank of mice. Tumor volumes were measured by length (a), width (b), and height (c), and calculated as: tumor volume = abc/2. When mouse tumor volumes reached 100–200 mm^3^, the mice were randomly divided into different groups. Tumor-bearing mice were treated with 15 Gy or 20 Gy tumor-localized IR (1 dose). For cGAMP treatment experiments, 10 μg 2′3′-cGAMP (InvivoGen, catalog tlrl-nacga23-1) was administered i.t. every other day for a total of 2 injections. For E64 treatment experiments, 50 μM E64 (MilliporeSigma, catalog 66701-25-5) was given daily via i.t. injection in volumes of 50 μL per mouse. For CD8^+^ T cell depletion, 200 μg anti-CD8 mAb (Leinco, catalog C375) was delivered twice weekly by i.p. injection, starting 1 day before IR. Mice were euthanized when the tumor volume reached 2,000 mm^3^ (according to the IACUC protocol).

### Flow cytometry.

Tumors were collected, excised, and diced followed by digestion into a cell suspension using digesting media containing 1 mg/mL collagenase type I (Worthington Biochemical, catalog LS004197) and 200 μg/mL DNase I (MilliporeSigma, catalog 10104159001). Digested samples were then passed through a 70 μm cell strainer and washed twice with staining buffer (PBS with 2% FBS and 0.5 mM EDTA). Cells were blocked with anti-FcR (BioXCell, catalog BE0307) for 10 minutes and subsequently stained with surface antibodies for 30 minutes at 4°C in the dark. Dead cells were marked using the Zombie UV Fixable Viability Kit (BioLegend, catalog 423107). For intracellular IFN-γ and granzyme B staining, cells were stimulated for 6 hours in vitro with Cell Activation Cocktail with Brefeldin A (BioLegend, catalog 423303). Cells were then permeabilized using the Fixation and Permeabilization Kit (BD, catalog 554714) and stained with IFN-γ APC–specific antibodies (BioLegend, catalog 505810) and Granzyme B Pacific Blue–specific antibodies (BioLegend, catalog 515408) for 30 minutes at 4°C in the dark. For intracellular staining of YTHDF1 and cathepsins A and B, the permeabilized cells were stained with YTHDF1 (Proteintech, catalog 17479-1-AP), cathepsin A (Proteintech, catalog 15020-1-AP), and cathepsin B (Cell Signaling Technology, catalog 31718) at 4°C overnight. The next day, cells were stained with Alexa Fluor 647 goat anti–rabbit IgG (Invitrogen, Thermo Fisher Scientific, catalog A21247) for 1 hour at room temperature. Samples were analyzed on a Cytek Aurora (Cytek Biosciences) at The University of Chicago Flow Cytometry Core facility, and data were analyzed using FlowJo software (Tree Star).

The following murine surface flow cytometry antibodies (all from BioLegend) were used: CD45 BV510 (catalog 103137), CD11c PE/cyanine 7 (catalog 117318), MHC-II AF700 (catalog 107622), CD80 Pacific Blue (catalog 104724), CD86 APC/cyanine 7 (catalog 105030), CD11b BV711 (catalog 101241), XCR1 BV650 (catalog 148220), PD-L1 APC (catalog 124312), and CD3 BV711 (catalog 100241). F4/80 BUV805 (catalog 749282) and CD8 BUV395 (catalog 563786) were from BD. H-2K^b^-SIINFEKL PE (catalog 12-5743-82) was from eBioscience.

The following human surface flow cytometry antibodies (all from BioLegend) were used: CD4 BV750 (catalog 344644), CD14 BV510 (catalog 301842), HLA-DR APC/Fire 750 (catalog 307658), and CD11c BV605 (catalog 301636). CD16 eFluor 450 (catalog 48-0168-42) was from Invitrogen. CD56 BUV563 (catalog 612929) was from BD. CD45 BUV805 (catalog 368-0459-42), CD3 AF532 (catalog 58-0038-42), and CD8 PerCP-eFluor 710 (catalog 46-0087-42) were from Thermo Fisher Scientific.

### ELISPOT assay.

For the DC functional assay, CD11c^+^ cells were purified with the EasySep Mouse CD11c Positive Selection Kit II (STEMCELL Technologies, catalog 18780) from TDLNs of B16-OZ tumor–bearing mice 5 days after IR or irradiated B16-OZ and BMDCs cocultures. OT-I naive CD8^+^ T cells were isolated from lymph nodes and spleen of naive OT-I transgenic mice with EasySep Mouse CD8^+^ T Cell Isolation Kit (STEMCELL Technologies, catalog 19853). CD11c^+^ cells (2 × 10^4^) were incubated with CD8^+^ T cells for 3 days at a ratio of 1:10. For the CD8^+^ T cell functional assay, CD8^+^ T cells were isolated from TDLNs of B16-OZ tumor–bearing mice 8 days after IR. CD8^+^ T cells (2 × 10^5^) were restimulated with 5 μg/mL SIINFEKL (InvivoGen, catalog vac-sin) and cocultured with 2 × 10^4^ naive CD11c^+^ cells for 2 days. The cytokine spots of IFN-γ were detected using an ELISPOT assay kit (BD, catalog 551083).

### ELISA.

For in vivo experiments, tumor tissues were collected on day 3 after IR and homogenized in RIPA (Thermo Fisher Scientific, catalog 89900) with a protease inhibitor (Thermo Fisher Scientific, catalog 78429). Homogenized tissues were centrifuged at 5,000*g* for 20 minutes to collect the supernatant. For in vitro experiments, 5 × 10^6^ B16-OZ cells were plated in 10 cm cell culture dishes overnight. The next day, B16-OZ cells were treated with 40 Gy and incubated for 12 hours. BMDCs (5 × 10^6^) were cocultured with IR-treated B16-OZ cells in the presence of fresh FLT3L for an additional 8 hours. After coculturing, CD11c^+^ cells were purified with an EasySep Mouse CD11c Positive Selection Kit II and seeded into 96-well plates (1 × 10^6^ cells/mL/well). Cell supernatants were harvested after a 2-day incubation. The concentration of IFN-β from tissue or CD11c^+^ cell supernatant was measured with a Mouse IFN-β ELISA Kit (PBL Assay Science, catalog 42410) in accordance with the manufacturer’s instructions.

### Real-time qPCR.

Total RNA was extracted using the RNeasy Plus Mini Kit (QIAGEN, catalog 74136). cDNA was then synthesized using a High-Capacity cDNA Reverse Transcription Kit (Thermo Fisher Scientific, catalog 4368814). Real-time qPCR (RT-qPCR) using SYBR Green PCR Master Mix (Thermo Fisher Scientific, catalog 43-687-02) was performed in QuantStudio 3 (Applied Biosystems) according to the manufacturer’s instruction. The specific primers for RT-qPCR are as follows: YTHDF1 forward, 5′-ACAGTTACCCCTCGATGAGTG-3′, YTHDF1 reverse, 5′-GGTAGTGAGATACGGGATGGGA-3′; IFN-β forward, 5′-ATGAGTGGTGGTTGCAGGC-3′, IFN-β reverse, 5′-TGACCTTTCAAATGCAGTAGATTCA-3′; ISG15 forward, 5′-CTAGAGCTAGAGCCTGCAG-3′, ISG15 reverse, 5′-AGTTAGTCACGGACACCAG-3′; IFIT3 forward, 5′-CCTACATAAAGCACCTAGATGGC-3′, IFIT3 reverse, 5′-ATGTGATAGTAGATCCAGGCGT-3′; CXCL10 forward, 5′-CCAAGTGCTGCCGTCATTTTC-3′, CXCL10 reverse, 5′-GGCTCGCAGGGATGATTTCAA-3′; GAPDH forward, 5′-AGGTCGGTGTGAACGGATTTG-3′, GAPDH reverse, 5′-TGTAGACCATGTAGTTGAGGTCA-3′; GAPDH was chosen as an endogenous control. Gene expression was calculated using the 2^–ΔΔCt^ method and is shown as the fold change versus the control.

### RIP–qPCR analysis.

RIP was performed with a Magna RIP RNA-Binding Protein Immunoprecipitation Kit (MilliporeSigma, catalog 17-700) in accordance with the manufacturer’s instructions. Briefly, BMDCs were lysed by RIP Lysis Buffer and sonicated. Anti-YTHDF1 antibody (Proteintech, catalog 17479-1-AP) or IgG control antibody (Cell Signaling Technology, catalog 2729) was incubated with magnetic beads for 30 minutes at room temperature to generate antibody-bead complexes. Cell lysates were then incubated with antibody-bead complexes overnight at 4°C. The next day, RNA was eluted and extracted via TRIzol (Thermo Fisher Scientific, 15596026). cDNA was then synthesized using the High-Capacity cDNA Reverse Transcription Kit (Thermo Fisher Scientific, catalog 4368814). RT-qPCR using SYBR Green PCR Master Mix (Thermo Fisher Scientific, catalog 43-687-02) was performed in QuantStudio 3 (Applied Biosystems).

### Western blot analysis.

Whole-cell protein was extracted with RIPA (Thermo Fisher Scientific, catalog 89900) containing protease (Thermo Fisher Scientific, catalog 78429) and phosphatase inhibitors (Thermo Fisher Scientific, catalog 78426). The protein concentration was measured with the Pierce BCA protein assay kit (Thermo Fisher Scientific, catalog 723225). Equal amounts of proteins were separated by SDS-PAGE and transferred onto PVDF membranes. Then, the membranes were blocked in 5% nonfat dry milk and probed with primary antibodies overnight at 4°C. The next day, the membranes were incubated with an HRP-conjugated secondary antibody after washing with TBST and finally detected using enhanced chemiluminescence (Thermo Fisher Scientific, catalog 32106). The primary antibodies used were STING (Proteintech, catalog 19851-1-AP), YTHDF1 (Proteintech, catalog 17479-1-AP), cathepsin A (Proteintech, catalog 15020-1-AP), and cathepsin B (Cell Signaling Technology, catalog 31718).

### Bulk RNA-Seq analysis.

BMDCs from WT and *Ythdf1*-cKO mice were cocultured with B16-OZ cells for 8 hours. The purified CD11c^+^ cells were incubated for an additional 2 days. RNA was extracted using an RNeasy Plus Mini Kit (QIAGEN, catalog 74136) and used to generate the complementary DNA library via a SMARTer Stranded Total RNA-Seq kit, version 2 (TaKaRa, catalog 634411). RNA-Seq was performed on an Illumina NovaSeq X platform by the Genomics Facility at The University of Chicago. We aligned reads using STAR (54) and removed alignments with a mapping quality of less than 30. We used DESeq2 (55) for differential gene expression analysis in which a negative binomial general linearized model fitting was used to generate *P* values, and *P* values were adjusted using the Benjamini-Hochberg method. GSEA version 4.3.2 was used for the rank-based test of enrichment performed using log-fold change values shrunken with the “apeglm” method from DESeq2.

### ChIP assay.

ChIP assays were performed with a Magna ChIP A/G Chromatin Immunoprecipitation Kit (MilliporeSigma, catalog 17-10086) in accordance with the manufacturer’s instructions. Briefly, 1 × 10^7^ BMDCs were treated with 100 ng/mL IFN-β1 for 1 hour and fixed with 1% formaldehyde, cross-linked, and sonicated. Cell lysates were incubated with anti-STAT2 antibody (Cell Signaling Technology, catalog 72604) or IgG control antibody (Cell Signaling Technology, catalog 2729) and protein A/G magnetic beads overnight at 4°C. The next day, protein/DNA complexes were eluted and reversed crosslinked. DNA was purified for RT-qPCR using the *Ythdf1* promoter DNA-specific primers. Input (1% of the chromatin) was chosen as the internal control and the results are shown as the percentage of input [100 × 2^([Input Ct – 6.64] – *Ythdf1* Ct)^].

### IP assay.

The IP assay was performed with a Dynabeads Protein G Immunoprecipitation Kit (Invitrogen, Thermo Fisher Scientific, catalog 10007D) in accordance with the manufacturer’s instructions. Briefly, anti-STING antibody (Proteintech, catalog 19851-1-AP) was incubated with magnetic dynabeads for 10 minutes at room temperature to generate antibody-conjugated magnetic beads. Cell lysates were then incubated with antibody-conjugated magnetic beads for 2 hours at room temperature to generate a bead-antibody-antigen complex. Target antigens were finally eluted from the bead-antibody-antigen complex and detected by Western blotting.

### Generation of the BMDC vaccine and in vivo vaccination.

The BMDC vaccine was generated as previously described (56, 57). Single-cell suspensions of bone marrow cells obtained from WT or *Ythdf1*-KO mice were cultured in RPMI-1640 medium (Gibco, Thermo Fisher Scientific, catalog 11875093) containing 10% FBS, 1% penicillin-streptomycin solution, and 20 ng/mL GM-CSF (BioLegend, catalog 576306) for 7 days. Fresh media with GM-CSF were added to the culture on days 3 and 5, and BMDCs were obtained on day 7. Immature DCs were pulsed with IR-treated B16-OVA cells at a ratio of 1:1 in the presence 250 ng/mL LPS (MilliporeSigma, catalog L2630) for 24 hours. CD11c^+^ cells were purified with the EasySep Mouse CD11c Positive Selection Kit II and used as the BMDC vaccine. C57BL/6 mice received a s.c. injection of 1 × 10^6^ B16-OVA cells on day 0. Fourteen days later (appearance of palpable tumors 100–200 mm^3^), tumor-bearing mice were treated with 20 Gy tumor-localized IR (1 dose). Antigen-pulsed BMDCs (3 × 10^6^) were injected i.t. twice weekly. For the BMDC vaccine generated with the YTHDF1 inhibitor SAC (MedchemExpress, catalog HY-N0319), immature DCs from WT mice were pulsed with IR-treated B16-OVA cells at a ratio of 1:1 in the presence 250 ng/mL LPS and treated or not with 20 μM SAC for 24 hours.

### Statistics.

Data were analyzed with GraphPad Prism 9 (GraphPad Software). Paired or unpaired, 2-tailed Student’s *t* tests were used to calculate the *P* values for comparisons between 2 groups, and 1-way ANOVA or 2-way ANOVA with Tukey’s multiple-comparison test was performed for multiple group comparisons. The survival curves were generated by the Kaplan-Meier method and compared using the log-rank (Mantel-Cox) test. The results are presented as the mean ± SEM unless otherwise noted. A *P* value of less than 0.05 was considered statistically significant.

### Study approval.

The COSINR study (NCT03223155) was approved by the University of Chicago Biological Sciences Division IRB (IRB 17-0547). Written informed consent was obtained from all patients. All experiments were performed in compliance with the Helsinki Declaration. The mouse study was approved by the IACUC of the University of Chicago (animal protocol no. 70931).

### Data availability.

Bulk RNA-Seq data are available from the NCBI’s Gene Expression Omnibus (GEO) database (GEO GSE272204). The expression correlation between IFNAR and YTHDF1 in this study was obtained from TIMER2.0 at http://timer.comp-genomics.org This study analyzed the existing scRNA-Seq and ChIP-Seq datasets from GEO at GSE206387and GSE115435, respectively (32, 34). Values for all data points in graphs are provided in the Supporting Data Values file.

## Author contributions

CW, LW, CH, HLL, and RRW designed the study. CW performed most of the experiments. XY, KZ, and MEM helped perform the bioinformatics analysis. AP performed the patient PBMC flow analysis. DC, JB, KY, EZN, FJ, JW, XH, SYL, LT, and BS helped perform animal experiments and in vitro experiments. SJC and SP provided clinical trial samples. CW, LW, HLL, and RRW wrote the manuscript. AP, JB, AA, CL, and CH helped edit the manuscript.

## Supplementary Material

Supplemental data

Unedited blot and gel images

Supporting data values

## Version 1 09/26/2024

In-Press Preview

## Version 2 12/02/2024

Electronic publication
