# Supplementary material for: YTHDF1 loss in dendritic cells potentiates radiation-induced antitumor immunity via STING-dependent type I IFN production
Source: J Clin Invest. 2024 Dec 2;134(23):e181612. doi: 10.1172/JCI181612 (PMC11601937; doi:10.1172/JCI181612)
Supplement: Supplemental data [file jci-134-181612-s186.pdf]

Supplemental Figures and Legends

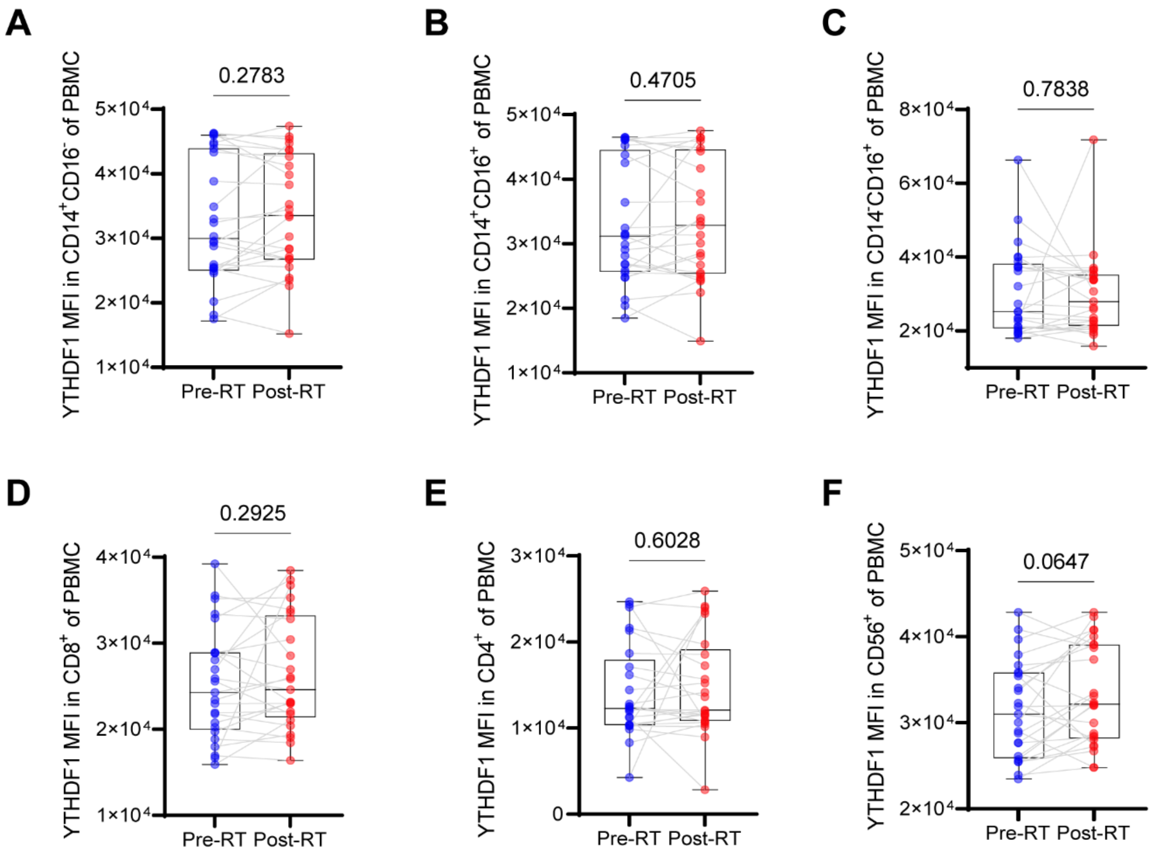

Supplemental Figure 1. Radiotherapy does not significantly increase YTHDF1 expression in monocytes, T cells and NK cells of patient PBMCs.

(A-F). Flow cytometry analysis of YTHDF1 expression in PBMCs from patients with metastatic NSCLC treated with SBRT in the following populations (n = 25): classical monocytes ($CD14^+CD16^-$) **(A)**; intermediate monocytes ($CD14^+CD16^+$) **(B)**; non-classical monocytes ($CD14^-CD16^+$) **(C)**; $CD8^+$ T cells ($CD3^+CD8^+$) **(D)**; $CD4^+$ T cells ($CD3^+CD4^+$) **(E)**; and NK cells ($CD3^-CD56^+$) **(F)**. Two-sided paired Student's *t*-test (A-F).

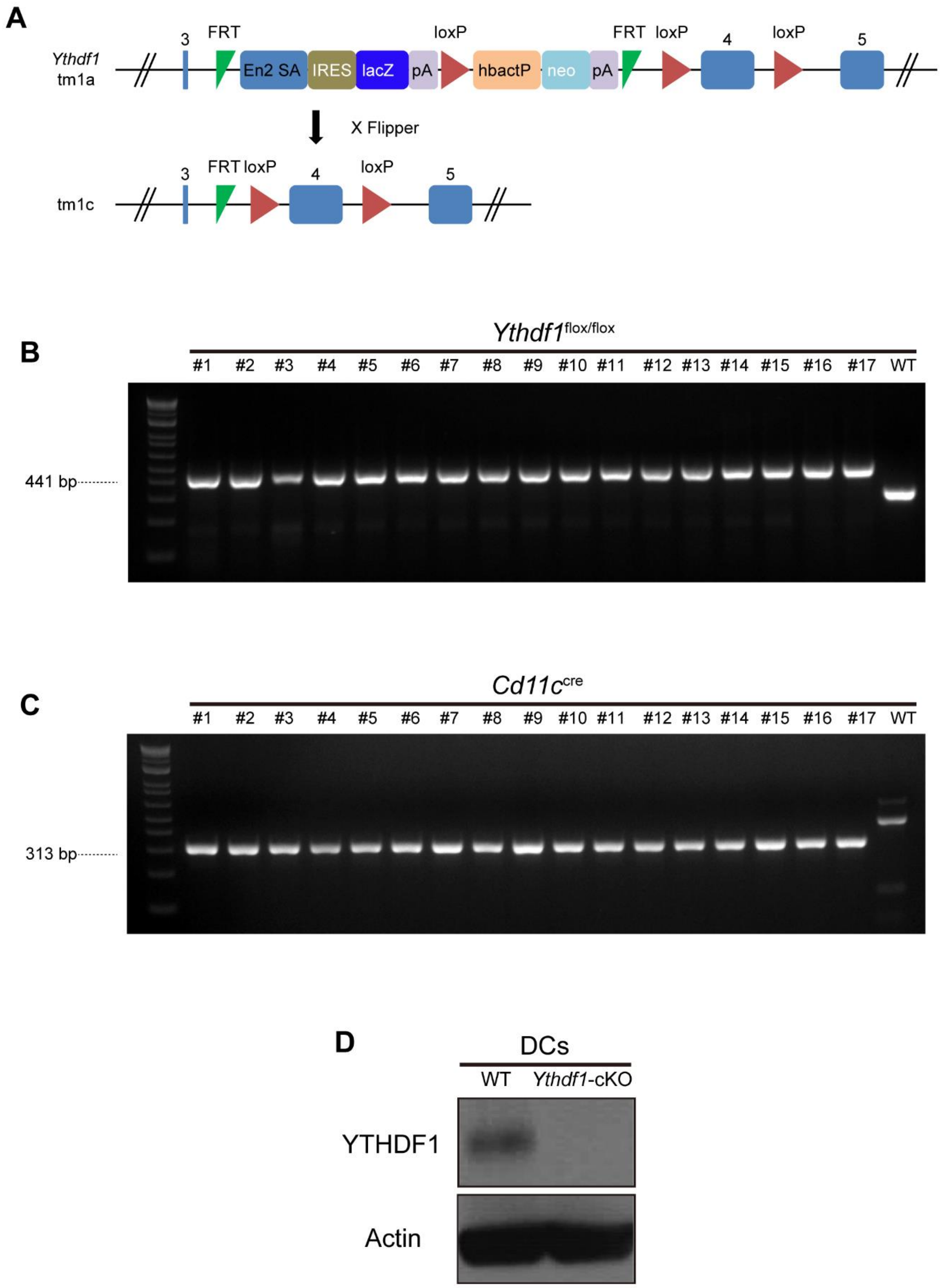

Supplemental Figure 2. Generation of mice with DC-specific *Ythdf1* deletion.

(A). The gene-targeting strategy for generating *Ythdf1*$^{flox/flox}$ mice.

(B and C). PCR products from *Ythdf1*$^{flox/flox}$ mice **(B)** and *Cd11c*^{Cre} mice **(C)**.

(D). Western blot analysis of the YTHDF1 expression in spleen DCs from WT and *Ythdf1*-cKO

mice.

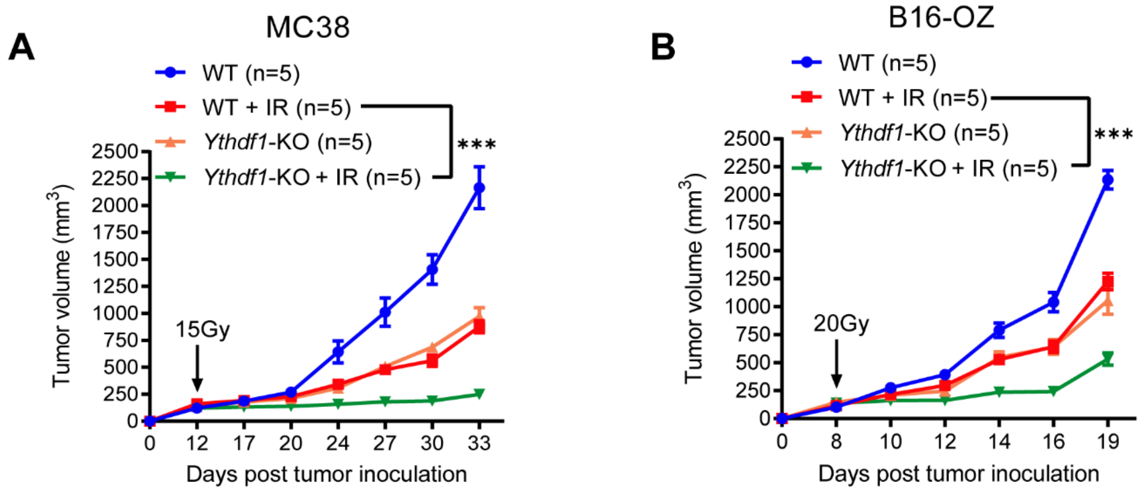

Supplemental Figure 3. *Ythdf1* depletion enhances the antitumor response to radiotherapy. (A and B). WT and *Ythdf1*-KO mice were injected subcutaneously with MC38 **(A)** and B16-OZ **(B)** cells. Tumor-bearing mice were treated with local IR (one dose) when tumor volume reached 100-200 mm^3. Tumor growth was monitored after IR.

Data are represented as mean ± SEM. Data are representative of three independent experiments. Two-way ANOVA with Tukey's multiple comparison test. ***P < 0.001.

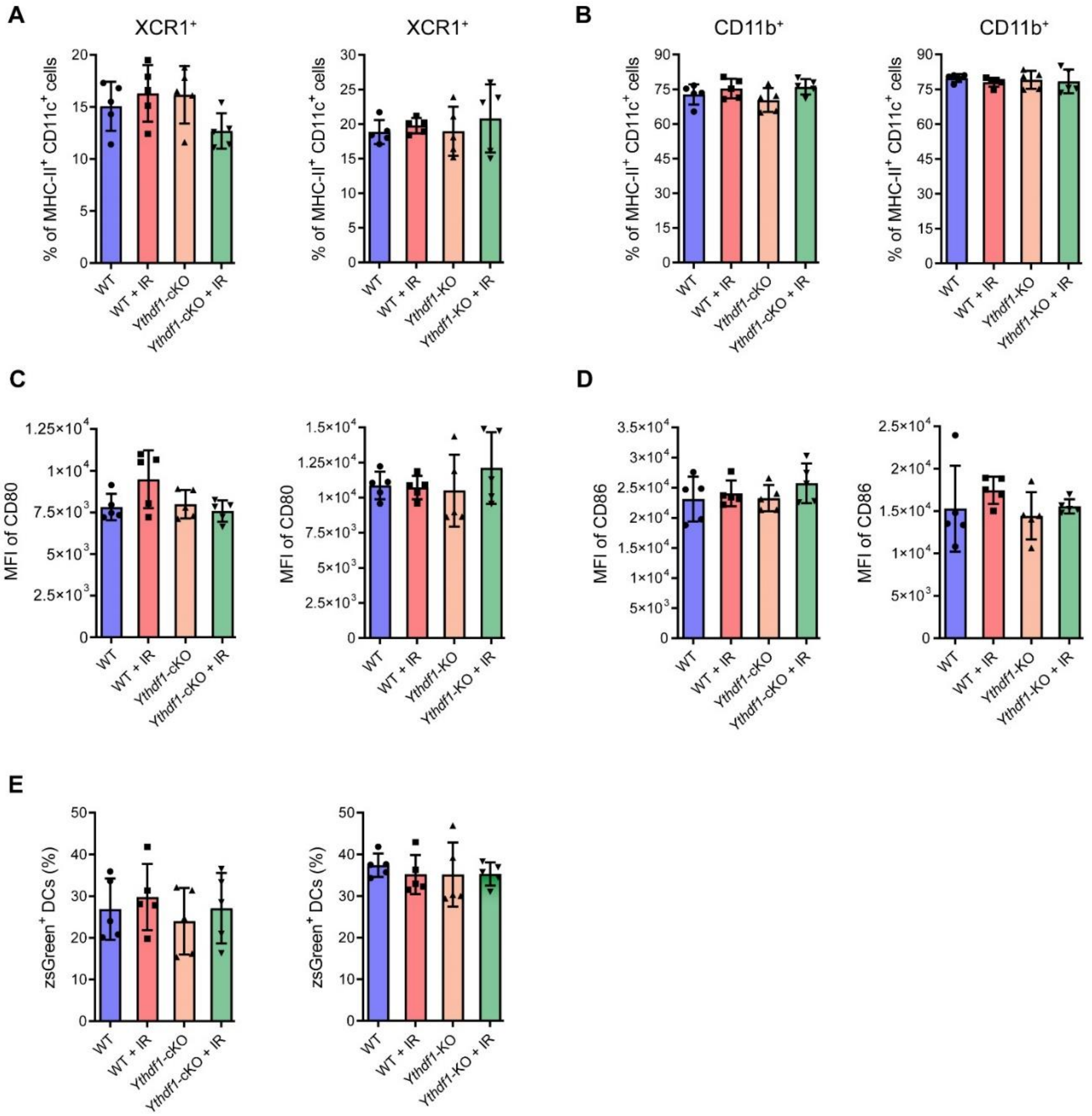

Supplemental Figure 4. IR has no significant effect on the subpopulation composition, co-stimulatory molecule expression, and phagocytic activity of tumor-infiltrating DCs in *Ythdf1*-cKO and *Ythdf1*-KO mice.

(A-E). *Ythdf1*-cKO and *Ythdf1*-KO mice were injected subcutaneously with B16-OZ cells. Tumor-bearing mice were treated with local IR (20 Gy, one dose) when tumor volume reached 100-200 mm^3. The following markers were detected via flow cytometry on day 5 after IR (n = 5).

DC1 (CD45$^+$F4/80$^-$CD11c$^+$MHC-II$^+$CD11b$^-$XCR1$^+$) **(A)** and DC2 (CD45$^+$F4/80$^-$CD11c$^+$MHC-II$^+$CD11b$^+$XCR1$^-$) **(B)** subpopulations of tumor-infiltrating DCs. The expression of CD80 **(C)** and CD86 **(D)** of tumor-infiltrating DCs (CD45$^+$F4/80$^-$CD11c$^+$MHC-II$^+$). The phagocytosis of tumor-infiltrating DCs were detected by the expression of zsGreen **(E)**.

Data are represented as mean ± SEM. Data are representative of two or three independent experiments. One-way ANOVA with Tukey's multiple comparison test (A-E).

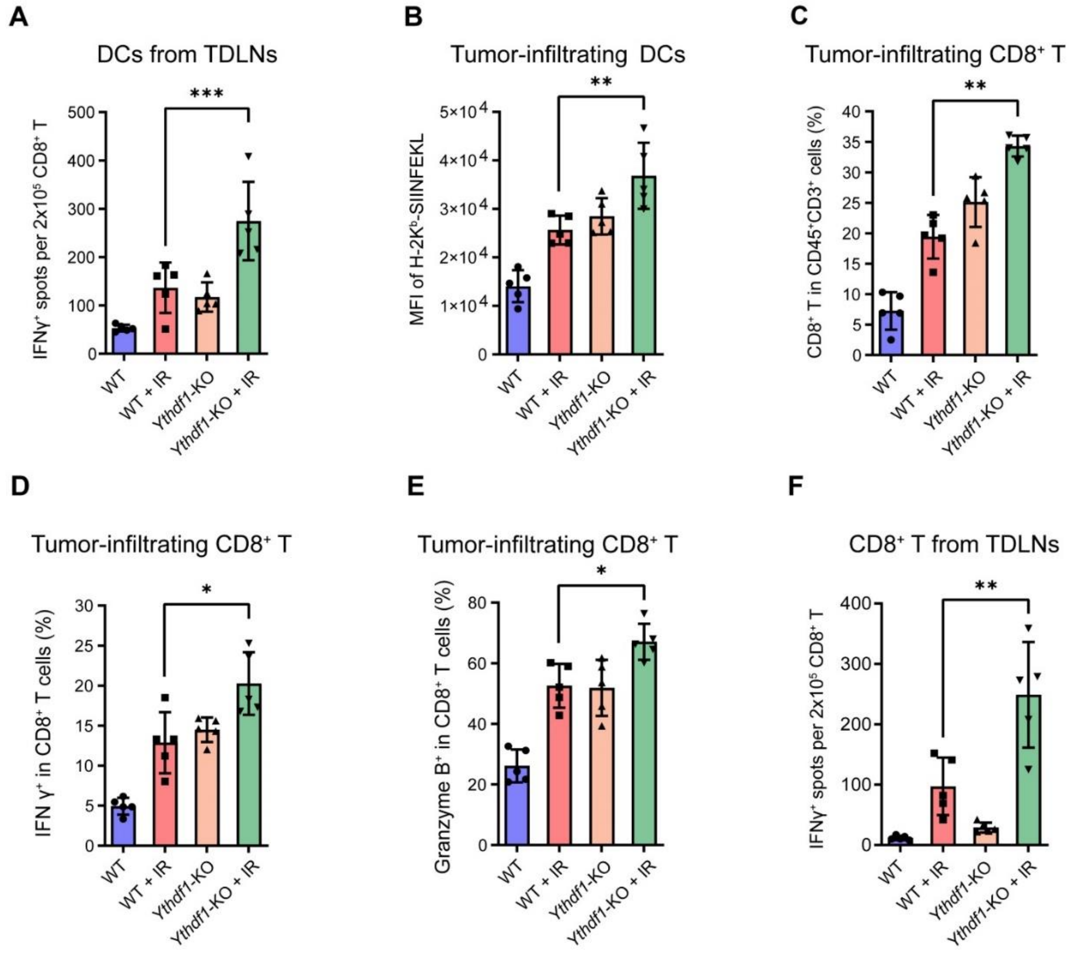

Supplemental Figure 5. *Ythdf1* whole body deletion enhances the cross-priming capacity of DCs in the context of IR.

(A and B). WT and *Ythdf1*-KO mice were injected subcutaneously with B16-OZ cells. Tumor-bearing mice were treated with local IR (20 Gy, one dose) when tumor volume reached 100-200 mm^3. On day 5 after IR, $CD11c^+$ cells from TDLNs were isolated and co-cultured with OT-I T cells for 3 days, and then IFNγ-producing cells were enumerated by ELISPOT (n = 5) **(A)**; in tumor-infiltrating DCs ($CD45^+F4/80^-CD11c^+MHC\text{-}II^+$), the formation of $H\text{-}2K^b$-SIINFEKL was detected via flow cytometry (n = 5) **(B)**.

(C-E). On day 8 post-IR, the proportions of $CD8^+$ T cells ($CD45^+CD3^+CD8^+$) **(C)**, IFNγ **(D)**, and Granzyme B **(E)** in $CD8^+$ T cells were detected via flow cytometry (n = 5).

(F). On day 8 post-IR, $CD8^+$ T cells were isolated from TDLNs. Tumor antigen-specific $CD8^+$ T-cell function was measured via ELISPOT by co-culturing $CD8^+$ T cells with 5 μg/ml OT-I peptide (n = 5).

Data are represented as mean ± SEM. Data are representative of two or three independent experiments. One-way ANOVA with Tukey's multiple comparison test (A-F). *$P < 0.05$, **$P < 0.01$, ***$P < 0.001$.

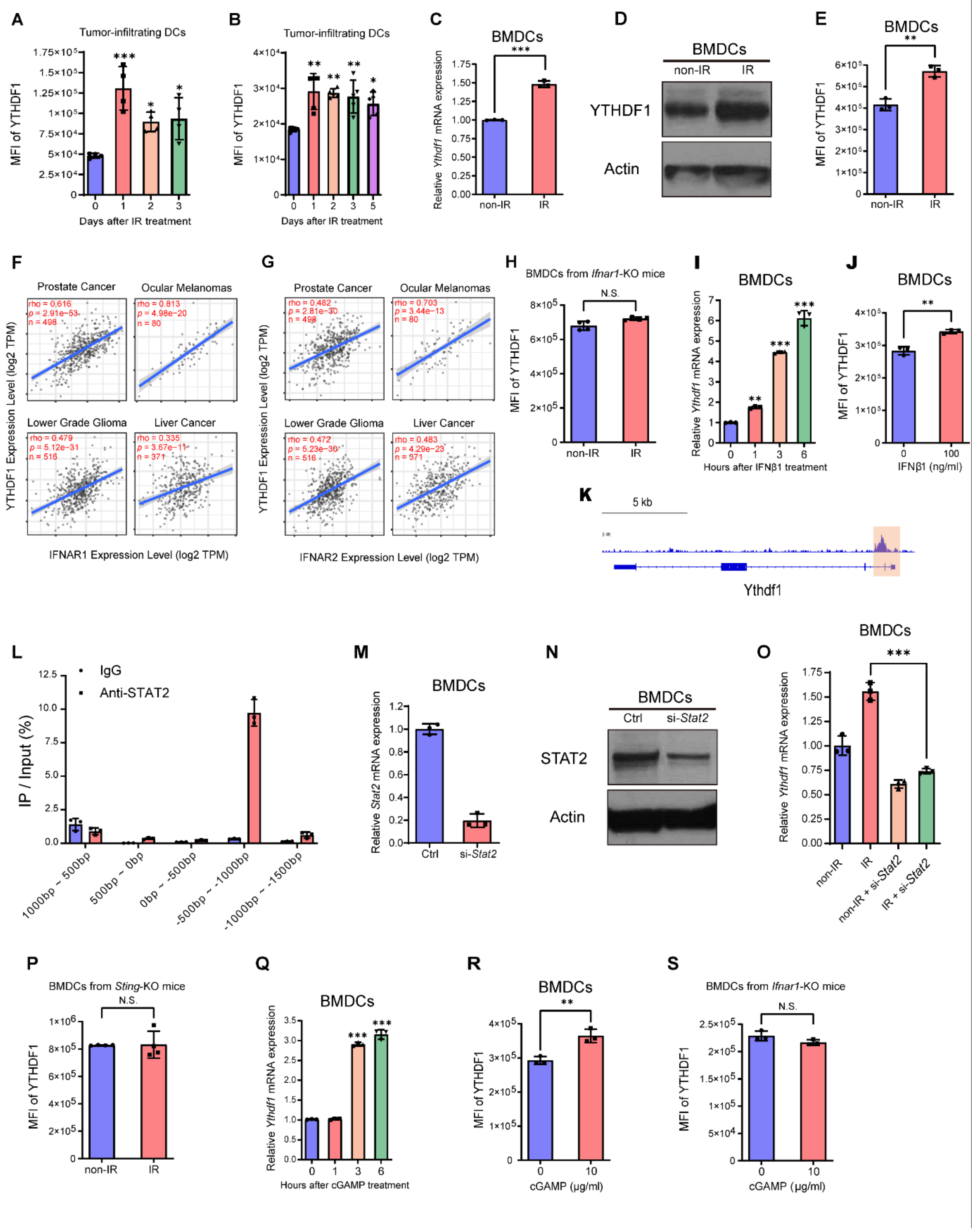

Supplemental Figure 6. IR induces YTHDF1 expression in DCs via STING-IFN-I signaling.

(A). B16-OZ-bearing mice were treated with local IR (20 Gy, one dose) and the expression of YTHDF1 of tumor-infiltrating DCs ($CD45^{+}F4/80^{-}CD11c^{+}MHC\text{-}II^{+}$) was detected at the indicated times after IR via flow cytometry (n = 5).

(B). B16-OZ-bearing mice were treated with local fractionated radiotherapy (5 Gy in 5 consecutive days) and the expression of YTHDF1 of tumor-infiltrating DCs ($CD45^{+}F4/80^{-}CD11c^{+}MHC\text{-}II^{+}$) was

detected at the indicated times after IR via flow cytometry (n = 5).

(C-E). BMDCs from WT mice were co-cultured with 40 Gy-pretreated B16-OZ or nonirradiated-B16-OZ cells, and the purified CD11c$^+$ cells were collected to detect *Ythdf1* mRNA expression by qPCR (co-cultured for 3 h, n = 3) **(C)**, and YTHDF1 protein expression by western blot (co-cultured for 24 h) **(D)**, and flow cytometry (co-cultured for 24 h, n = 3) **(E)**.

(F and G). The correlation between IFNAR1 **(F)** or IFNAR2 **(G)** and YTHDF1 expression was evaluated by Spearman's method.

(H). BMDCs from *Ifnar1*-KO mice were cultured with 40 Gy-pretreated B16-OZ or nonirradiated-B16-OZ cells for 24 h. The expression of YTHDF1 in BMDCs was then detected via flow cytometry (n = 4).

(I). BMDCs from WT mice were treated with 100 ng/ml IFNβ1. BMDCs cells were then collected at the indicated times to measure *Ythdf1* expression by qPCR (n = 3).

(J). BMDCs from WT mice were treated with 100 ng/ml IFNβ1 for 24 h. The expression of YTHDF1 was then detected by flow cytometry (n = 3).

(K). Profile of STAT2 binding (GSE115435) at the promoter region of *Ythdf1* in bone marrow-derived macrophages.

(L). STAT2 binding to the promoter region of *Ythdf1* was determined by ChIP-qPCR (n = 3).

(M and N). BMDCs were transfected with si-RNA targeting *Stat2.* Cells were then collected to detect *Stat2* mRNA expression by qPCR (24 h after transfection, n = 3) **(M)**, and YTHDF1 protein expression by western blot (48 h after transfection) **(N)**.

(O). BMDCs with STAT2 knockdown were co-cultured with 40 Gy-pretreated B16-OZ or nonirradiated-B16-OZ cells, and the purified CD11c$^+$ cells were collected to detect *Ythdf1* mRNA expression by qPCR (co-cultured for 3 h, n = 3).

(P). BMDCs from *Sting*-KO mice were co-cultured with 40 Gy-pretreated B16-OZ or nonirradiated-B16-OZ cells for 24 h. The expression of YTHDF1 was then detected by flow cytometry (n = 4).

(Q). BMDCs from WT mice were treated with 10 μg/ml 2'3'-cGAMP. BMDCs cells were then collected at the indicated times to measure *Ythdf1* expression by qPCR (n = 3).

(R). BMDCs from WT mice were treated with 10 μg/ml 2'3'-cGAMP for 24 h. The expression of YTHDF1 was then detected by flow cytometry (n = 3).

(S). BMDCs from *Ifnar1*-KO mice were treated with 10 μg/ml 2'3'-cGAMP for 24 h. The expression of YTHDF1 was then detected by flow cytometry (n = 3).

Data are represented as mean ± SEM. Data are representative of two or three independent experiments (A-E, H-J, L-S). Two-sided unpaired Student's *t*-tests (C, E, H, J, P, R and S), one-way ANOVA with Tukey's multiple comparison test (A, B, I, O and Q). N.S. = not significant, **P* < 0.05, ***P* < 0.01, ****P* < 0.001.

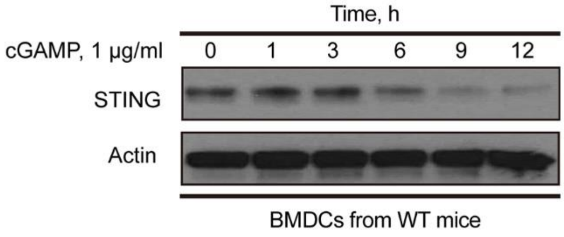

Supplemental Figure 7. 2'3'-cGAMP induces STING degradation in BMDCs.

BMDCs from WT mice were treated with 2’3’-cGAMP at the indicated times and the expression of STING was detected by western blot. Data are representative of three independent experiments.

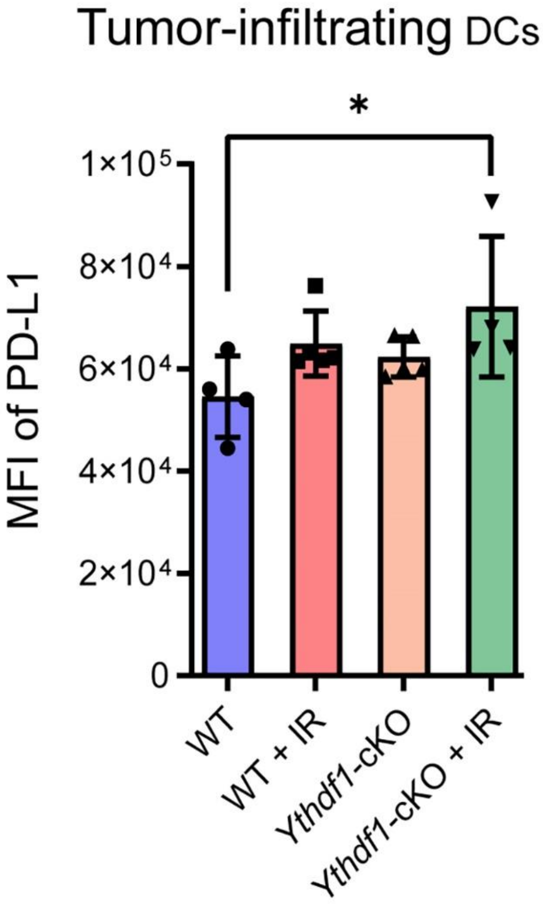

Supplemental Figure 8. The PD-L1 expression of tumor-infiltrating DCs is increased in *Ythdf1*-cKO+IR mice.

WT and *Ythdf1*-cKO mice were injected subcutaneously with B16-OZ cells. Tumor-bearing mice were treated with local IR (20 Gy, one dose) when tumor volume reached 100-200 mm^3. The expression of PD-L1 on tumor-infiltrating DCs ($CD45^+F4/80^-CD11c^+MHC\text{-}II^+$) was detected via flow cytometry on day 5 after IR (n = 4 or 5).

Data are represented as mean ± SEM. One-way ANOVA with Tukey’s multiple comparison test. *$P < 0.05$.
