# Supplementary material for: YTHDF1 loss in dendritic cells potentiates radiation-induced antitumor immunity via STING-dependent type I IFN production
Source: J Clin Invest. 2024 Dec 2;134(23):e181612. doi: 10.1172/JCI181612 (PMC11601937; doi:10.1172/JCI181612)
Supplement: Unedited blot and gel images [file jci-134-181612-s187.pdf]

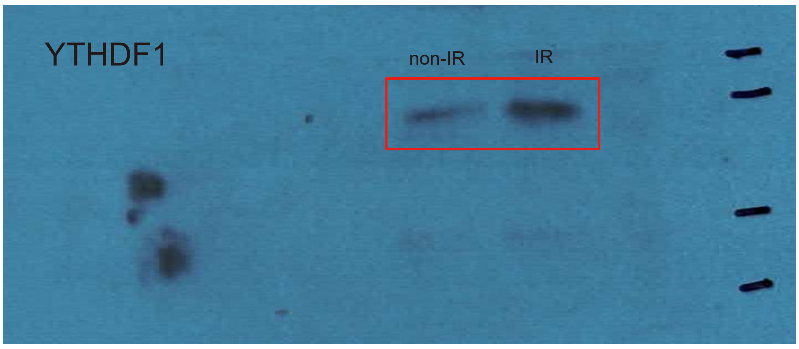

Full unedited gel for Figure 3C

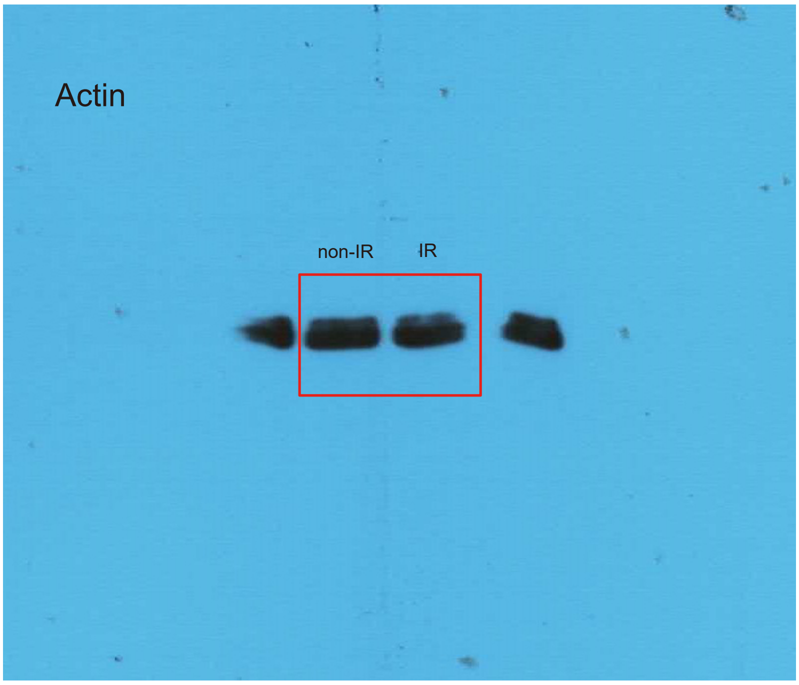

Full unedited gel for Figure 3C

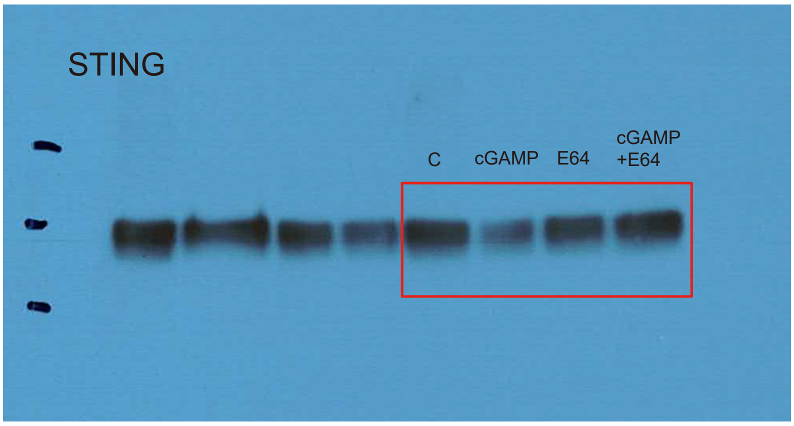

Full unedited gel for Figure 5C

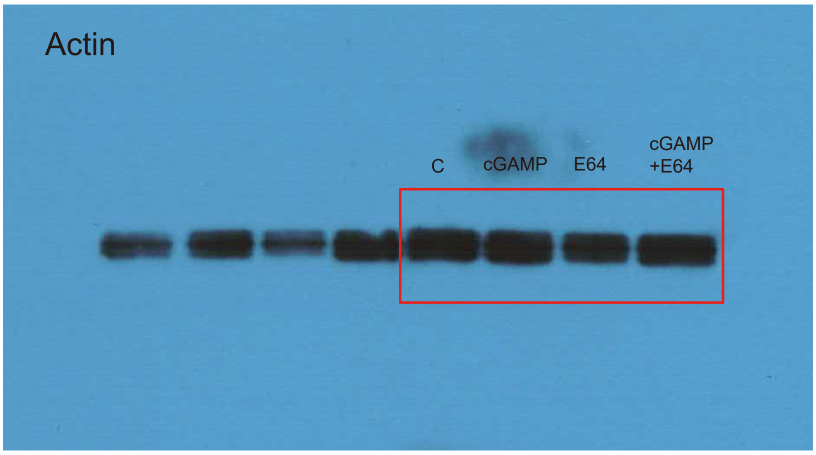

Full unedited gel for Figure 5C

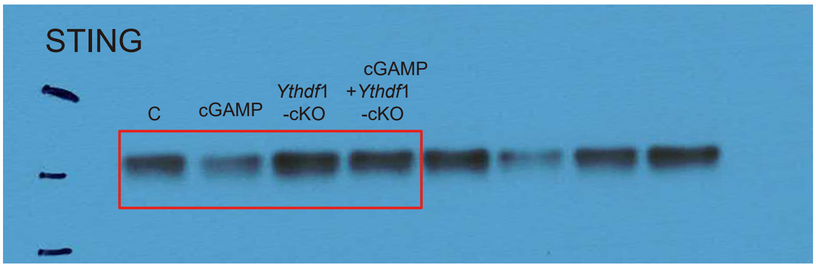

Full unedited gel for Figure 5D

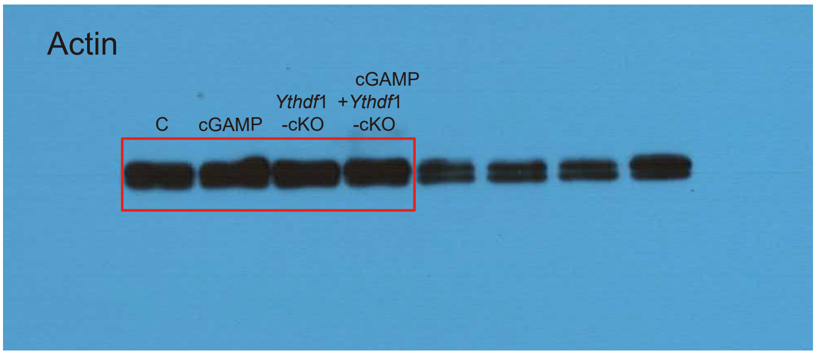

Full unedited gel for Figure 5D

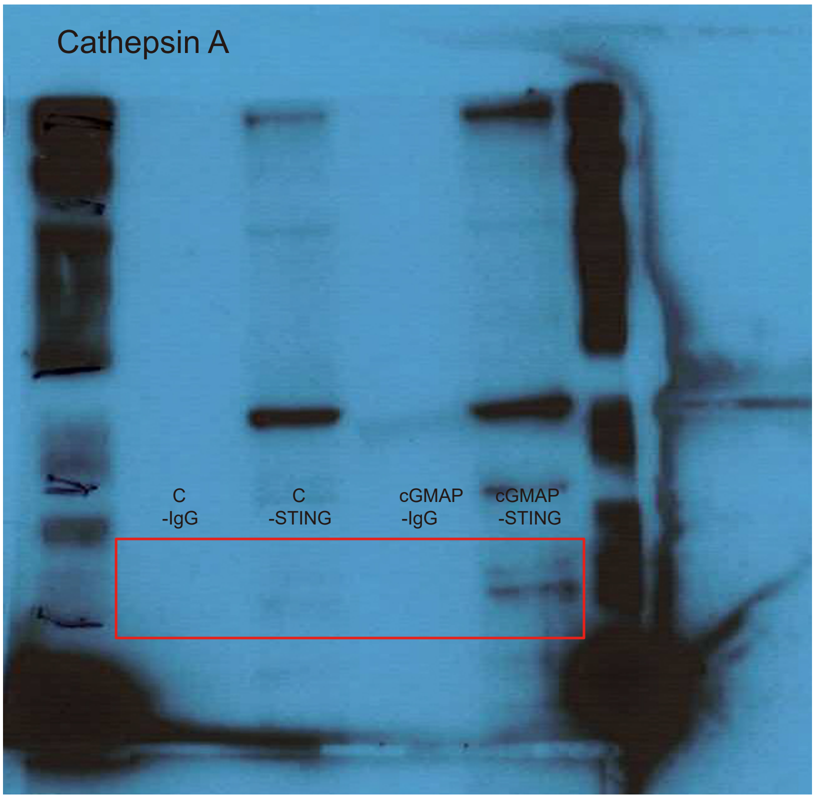

Full unedited gel for Figure 5E

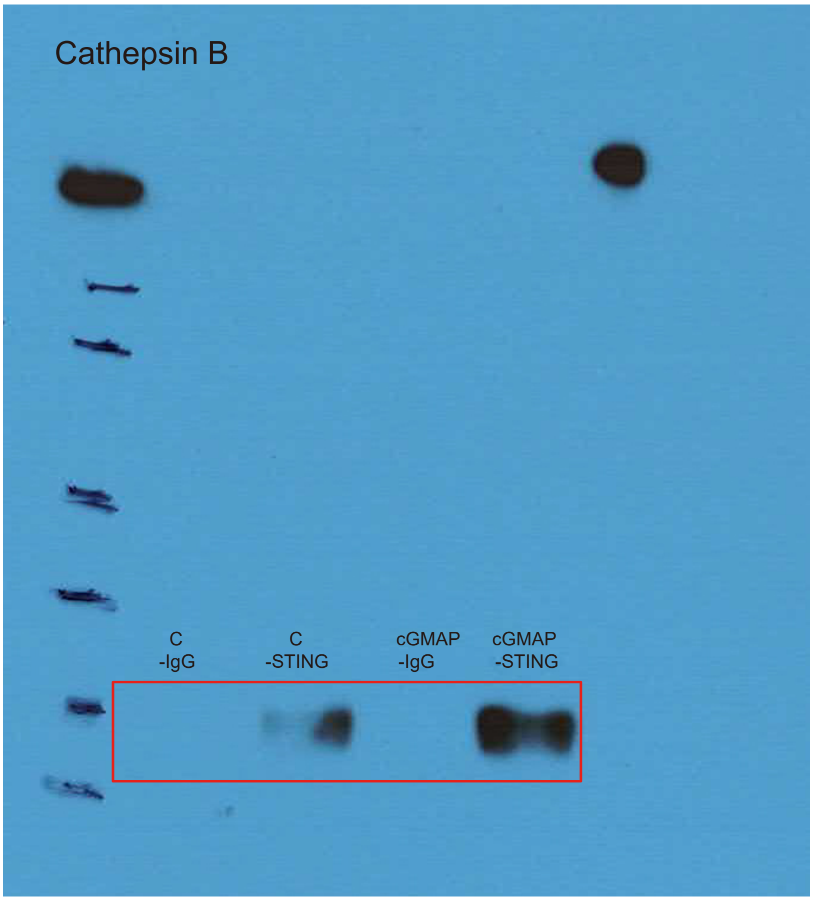

Full unedited gel for Figure 5E

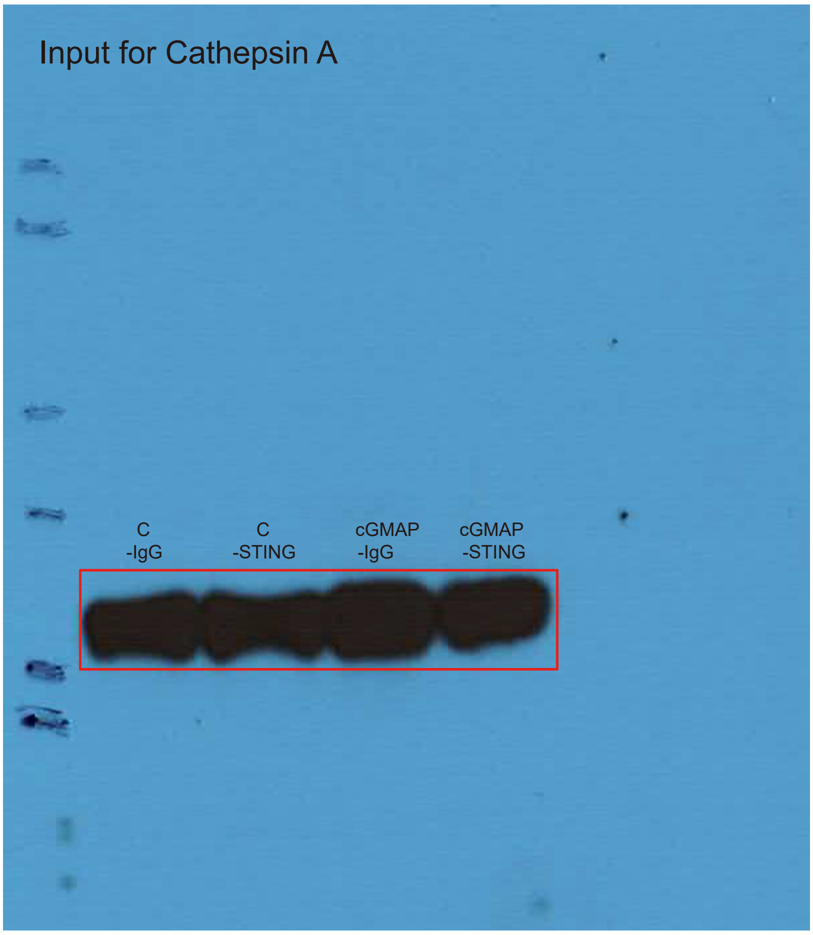

Full unedited gel for Figure 5E

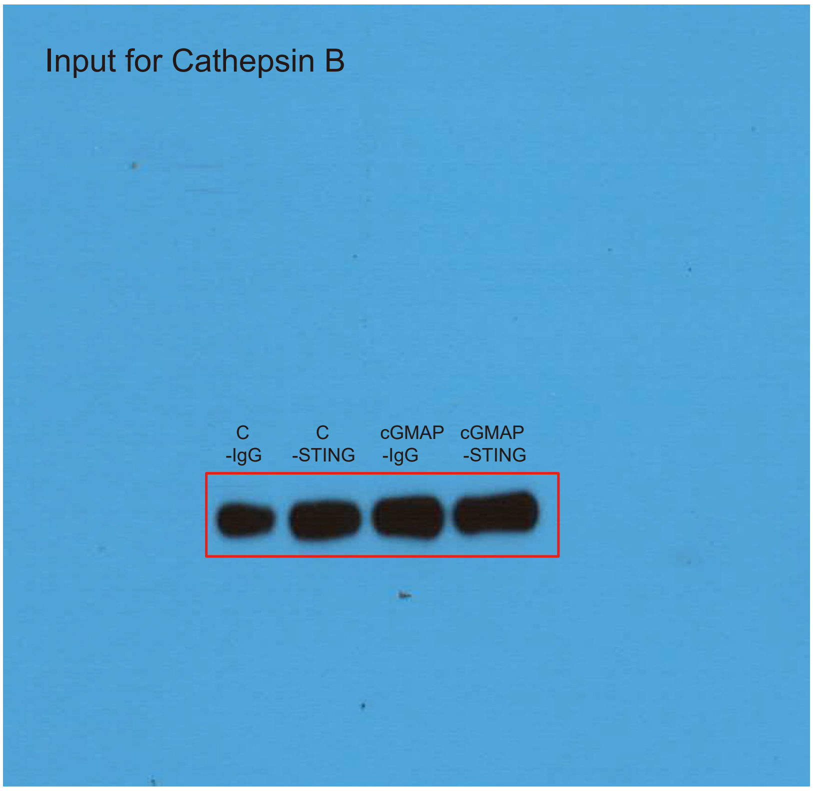

Full unedited gel for Figure 5E

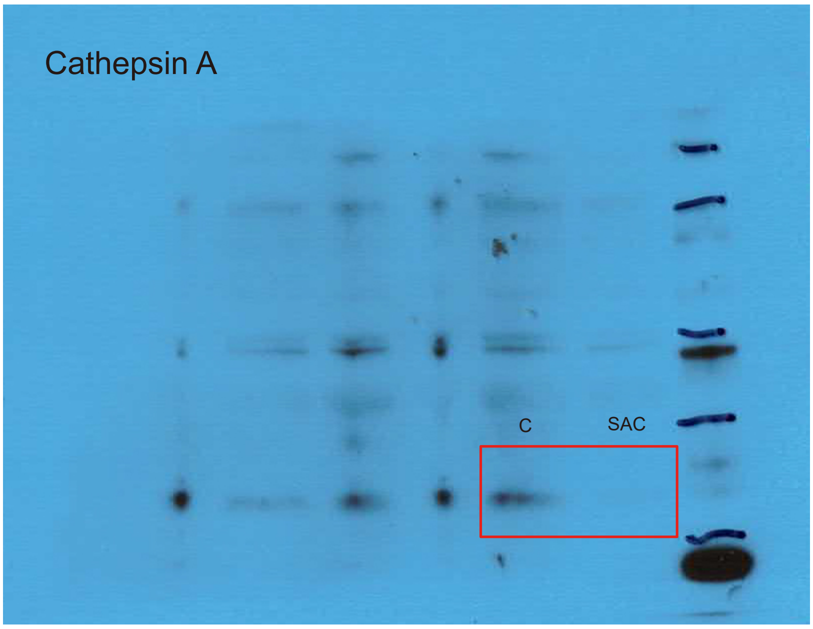

Full unedited gel for Figure 6C

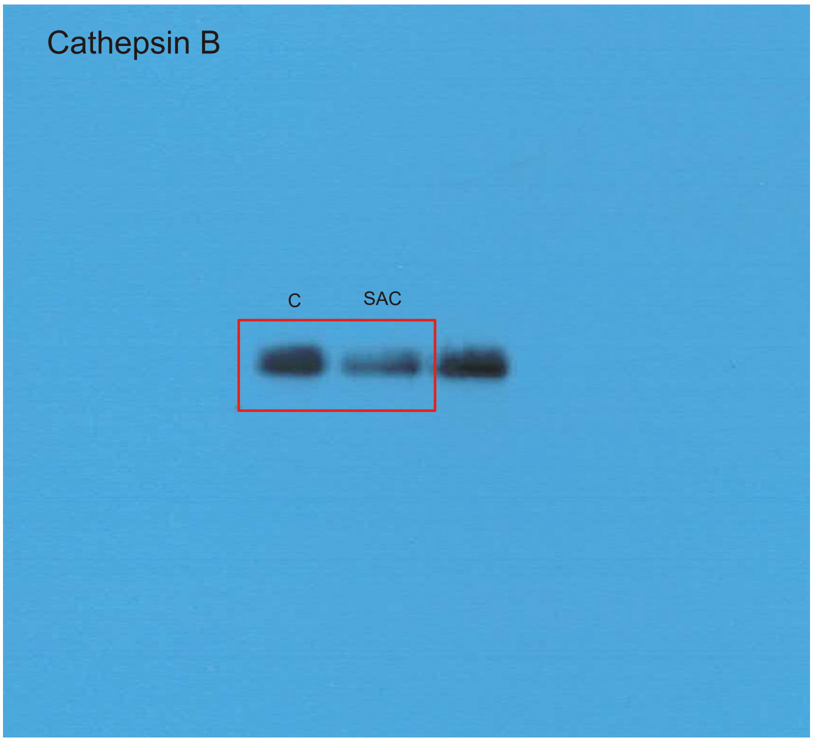

Full unedited gel for Figure 6C

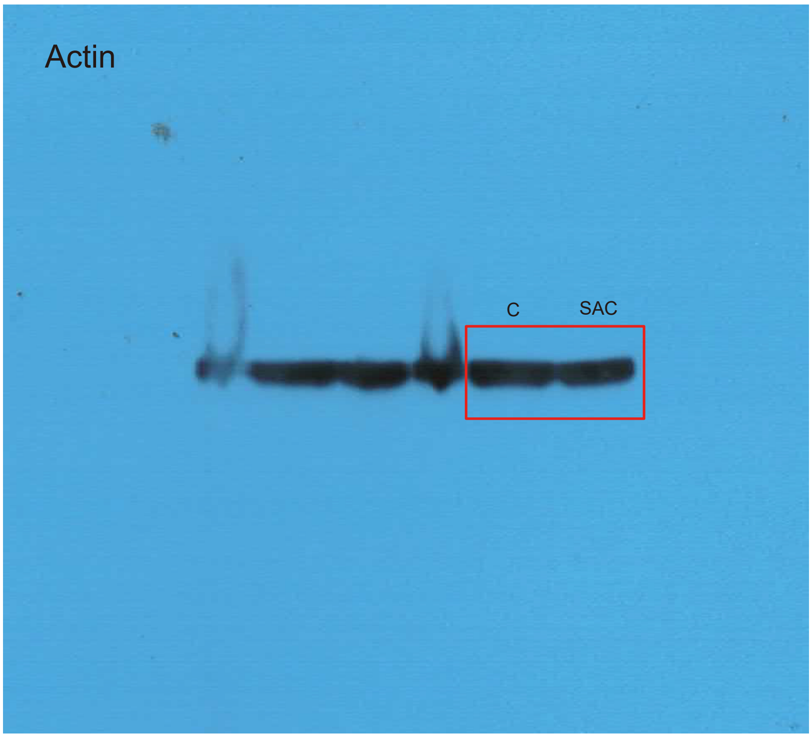

Full unedited gel for Figure 6C

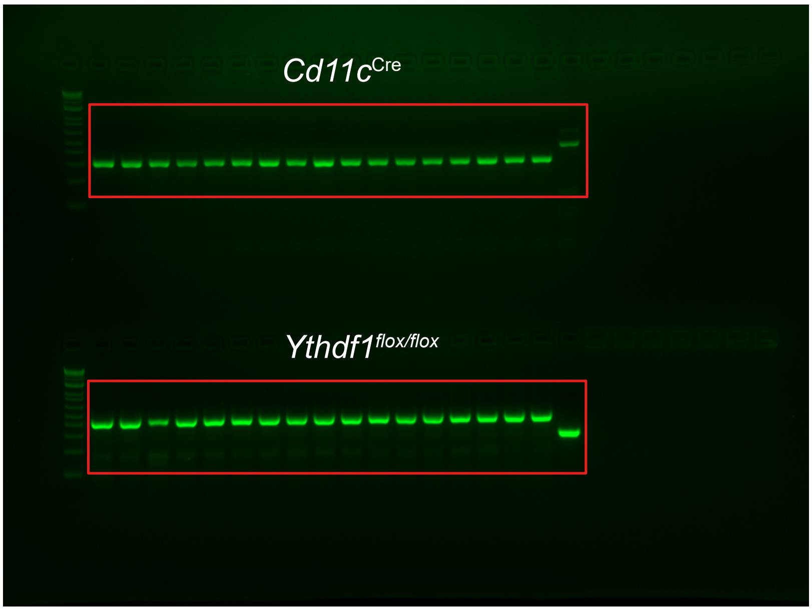

Full unedited gel for Supplemental Figure 2B and 2C

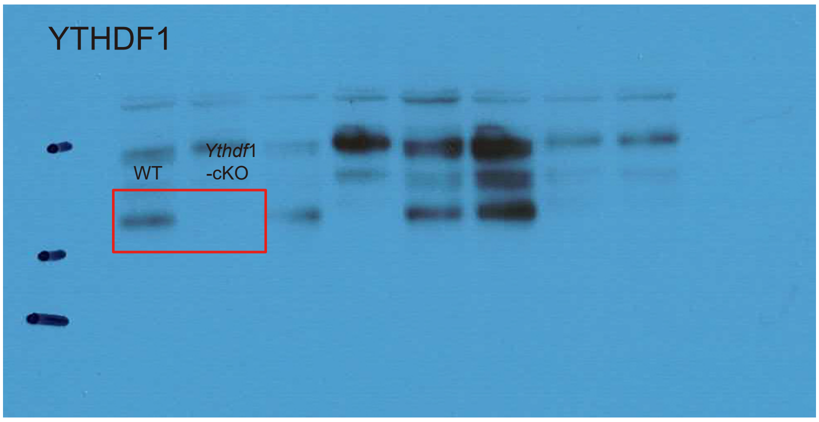

Full unedited gel for Supplemental Figure 2D

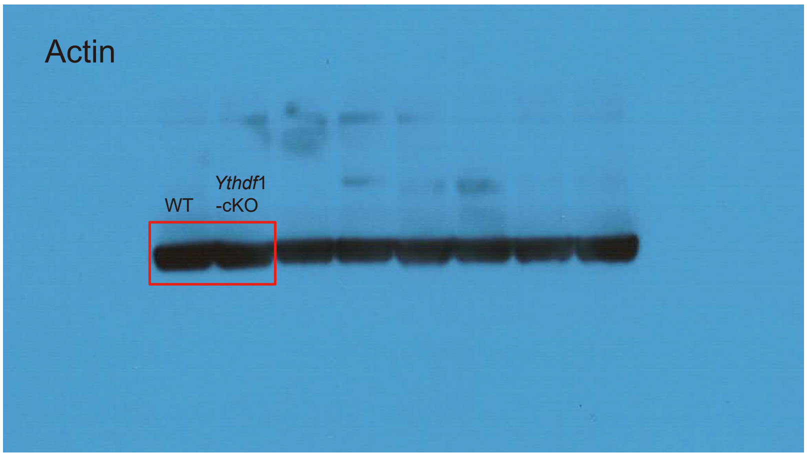

Full unedited gel for Supplemental Figure 2D

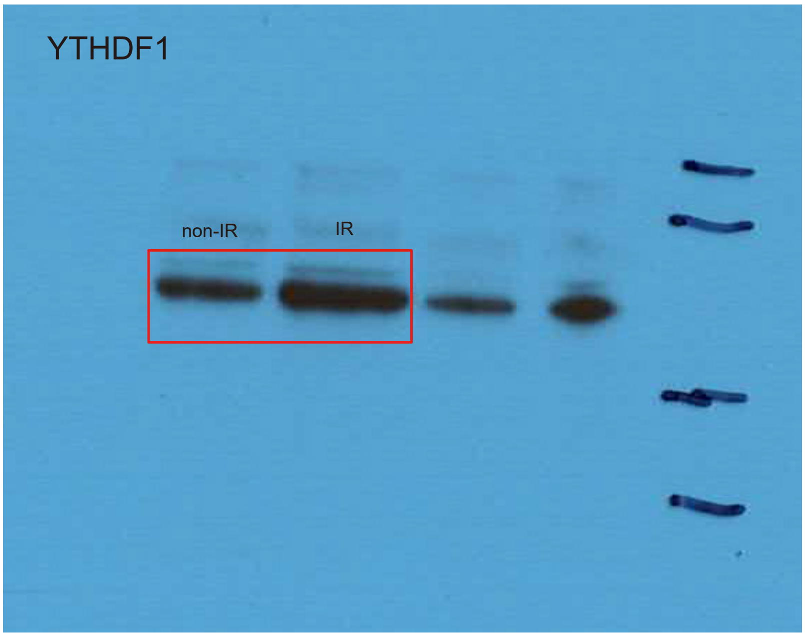

Full unedited gel for Supplemental Figure 6D

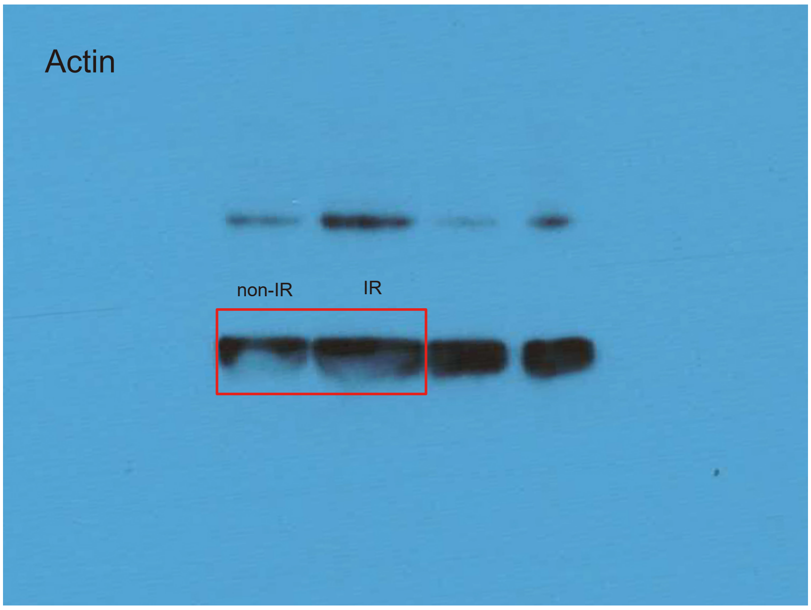

Full unedited gel for Supplemental Figure 6D

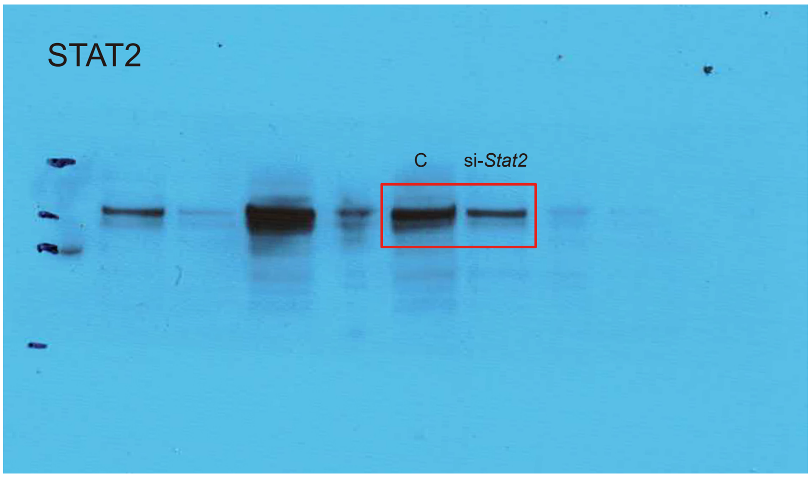

Full unedited gel for Supplemental Figure 6N

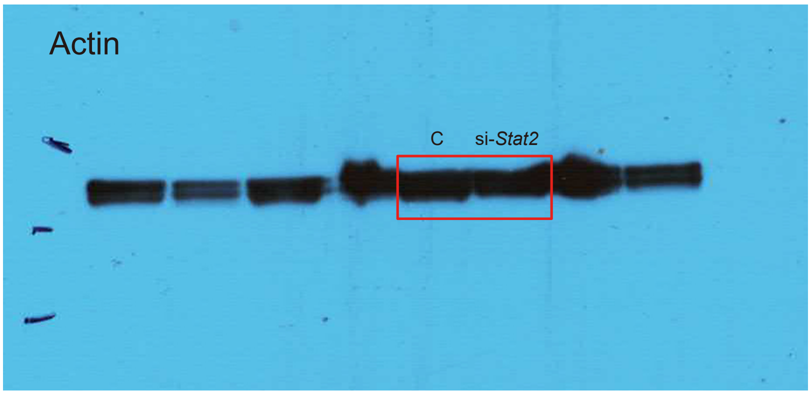

Full unedited gel for Supplemental Figure 6N

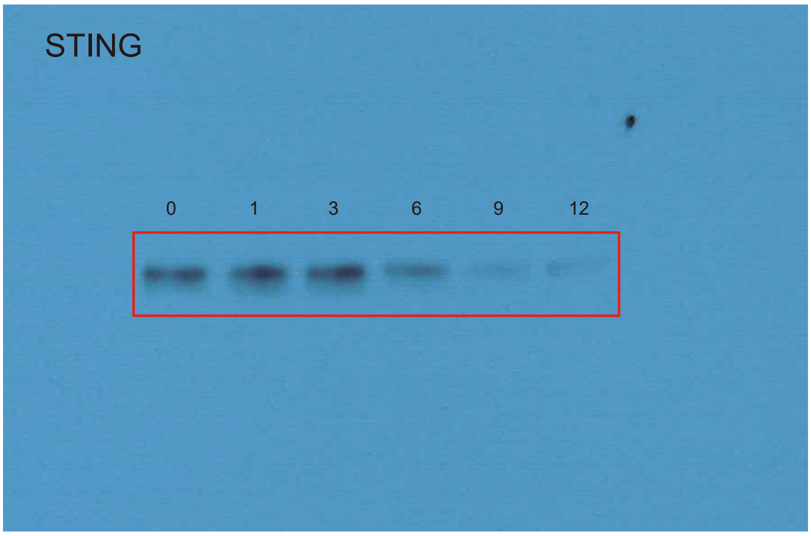

Full unedited gel for Supplemental Figure 7

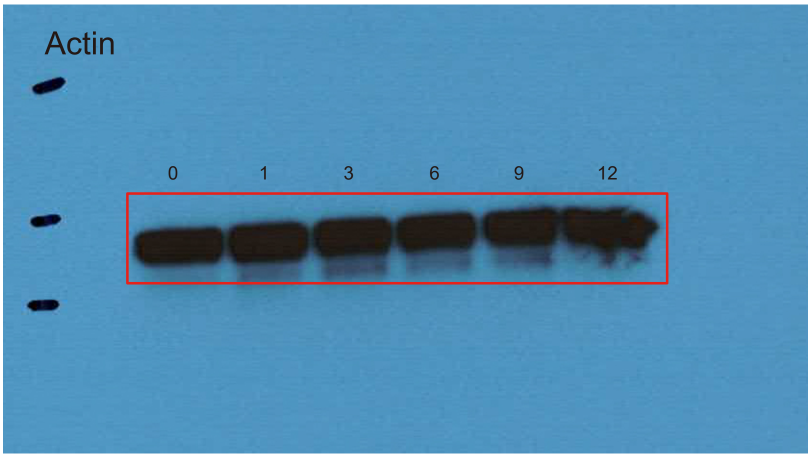

Full unedited gel for Supplemental Figure 7
